# Biogenic volatile organic compound ambient mixing ratios and emission rates in the Alaskan Arctic tundra

**DOI:** 10.5194/bg-17-6219-2020

**Published:** 2020-12-09

**Authors:** Hélène Angot, Katelyn McErlean, Lu Hu, Dylan B. Millet, Jacques Hueber, Kaixin Cui, Jacob Moss, Catherine Wielgasz, Tyler Milligan, Damien Ketcherside, M. Syndonia Bret-Harte, Detlev Helmig

**Affiliations:** 1 Institute of Arctic and Alpine Research, University of Colorado Boulder, Boulder, CO, USA; 2 Department of Chemistry and Biochemistry, University of Montana, Missoula, MT, USA; 3 Department of Soil, Water, and Climate, University of Minnesota, Minneapolis–Saint Paul, MN, USA; 4 Institute of Arctic Biology, University of Alaska Fairbanks, Fairbanks, Alaska, USA

## Abstract

Rapid Arctic warming, a lengthening growing season, and the increasing abundance of biogenic volatile-organic-compound-emitting shrubs are all anticipated to increase atmospheric biogenic volatile organic compounds (BVOCs) in the Arctic atmosphere, with implications for atmospheric oxidation processes and climate feedbacks. Quantifying these changes requires an accurate understanding of the underlying processes driving BVOC emissions in the Arctic. While boreal ecosystems have been widely studied, little attention has been paid to Arctic tundra environments. Here, we report terpenoid (isoprene, monoterpenes, and sesquiterpenes) ambient mixing ratios and emission rates from key dominant vegetation species at Toolik Field Station (TFS; 68°38′ N, 149°36′ W) in northern Alaska during two back-to-back field campaigns (summers of 2018 and 2019) covering the entire growing season. Isoprene ambient mixing ratios observed at TFS fell within the range of values reported in the Eurasian taiga (0–500 parts per trillion by volume – pptv), while monoterpene and sesquiterpene ambient mixing ratios were respectively close to and below the instrumental quantification limit (~ 2 pptv). Isoprene surface emission rates ranged from 0.2 to 2250 μgC m^−2^ h^−1^ (mean of 85 μgC m^−2^ h^−1^) and monoterpene emission rates remained, on average, below 1 μgC m^−2^ h^−1^ over the course of the study. We further quantified the temperature dependence of isoprene emissions from local vegetation, including *Salix* spp. (a known isoprene emitter), and compared the results to predictions from the Model of Emissions of Gases and Aerosols from Nature version 2.1 (MEGAN2.1). Our observations suggest a 180 %–215 % emission increase in response to a 3–4°C warming, and the MEGAN2.1 temperature algorithm exhibits a close fit with observations for enclosure temperatures in the 0–30°C range. The data presented here provide a baseline for investigating future changes in the BVOC emission potential of the under-studied Arctic tundra environment.

## 1 Introduction

As a major source of reactive carbon to the atmosphere, biogenic volatile organic compounds (BVOCs) emitted from vegetation play a significant role in global carbon and oxidation cycles (Fehsenfeld et al., 1992). Global emission estimates of BVOCs are in the range of 700–1100 TgC yr^−1^, ~ 70 %–80 % of which corresponds to terpenoid species, namely isoprene, monoterpenes (MTs), and sesquiterpenes (SQTs; Guenther et al., 1995, 2006; Sindelarova et al., 2014). Despite their relatively short atmospheric lifetimes (a few minutes to 1 d for terpenoids), BVOCs affect climate through their effects on the hydroxyl radical (OH, which dictates the lifetime of atmospheric methane), tropospheric ozone (O_3_, a key greenhouse gas), and aerosols (which influence radiative scattering) (Arneth et al., 2010; Fuentes et al., 2000; Peñuelas and Staudt, 2010). The oxidation of those BVOCs also drives the formation of secondary organic aerosols (SOAs) through both gas- and aqueous-phase mechanisms (Carlton et al., 2009; Lim et al., 2005). The potential for increased SOA formation, expected to result in climate cooling (Kulmala et al., 2004), complicates the climate feedbacks of BVOC emissions (Tsigaridis and Kanakidou, 2007; Unger, 2014).

Global models of BVOC emissions assume minimal emissions from the Arctic due to low leaf area index and relatively cold temperatures (Guenther et al., 2006; Sindelarova et al., 2014). However, this assumption relies on few observations and has been increasingly challenged by field data (Tang et al., 2016). Recent measurements have revealed significant BVOC emissions from Arctic tundra and vegetation, including *Sphagnum* mosses, wetland sedges, and dwarf shrubs (Ekberg et al., 2009, 2011; Faubert et al., 2010; Holst et al., 2010; Lindfors et al., 2000; Potosnak et al., 2013; Rinnan et al., 2011; Schollert et al., 2014; Tiiva et al., 2008). These results are of importance because BVOC emissions are expected to increase in the Arctic due to climate warming and associated vegetation and land cover change (Faubert et al., 2010; Potosnak et al., 2013; Rinnan et al., 2011; Tiiva et al., 2008). Field warming studies have shown strong increases in BVOC emissions from shrub heath (Michelsen et al., 2012; Tiiva et al., 2008). Furthermore, the temperature dependence of Arctic BVOC fluxes appears to be significantly greater than for tropical and subtropical ecosystems (Holst et al., 2010; Rinnan et al., 2014), with up to two-fold increases in MT emissions and five-fold increases in SQT emissions by subarctic heath for a 2 °C warming (Valolahti et al., 2015). Similarly, Kramshøj et al. (2016) and Lindwall et al. (2016) examined the response of BVOC emissions to an experimental 3–4 °C warming and reported a 260 %–280 % increase in total emissions. Together, the above results emphasize the strong temperature sensitivity of BVOC emissions from Arctic ecosystems.

Changing BVOC emissions in the Arctic due to climate and land cover shifts can thus be expected to perturb the overall oxidative chemistry of the region. Previous studies have hypothesized that BVOCs might already impact the diurnal cycle of ozone in the Arctic boundary layer (Van Dam et al., 2016). Changing BVOC emissions can also further affect climate through various feedback mechanisms. Quantifying these changes requires an accurate understanding of the underlying processes driving BVOC emissions in the Arctic. While BVOC ambient mixing ratios and emission rates have been studied in boreal ecosystems, less attention has been paid to Arctic tundra environments (Lindwall et al., 2015). Here, we report BVOC ambient mixing ratios and emission rates at Toolik Field Station (TFS) in the Alaskan Arctic. This study builds on the previous isoprene study at TFS by Potosnak et al. (2013), while also providing a major step forward from that work. In particular, we present the first continuous summertime record of ambient BVOCs (including isoprene and MT) and their first-generation oxidation products in the Arctic tundra environment. The data presented here provide a baseline for investigating future changes in the BVOC emission potential of the under-studied Arctic tundra environment. Due to increasing shrub prevalence across northern Alaska (Berner et al., 2018; Tape et al., 2006), and the Eurasian (Macias-Fauria et al., 2012) and Russian Arctic (Forbes et al., 2010), the results of this study have significance to tundra ecosystems across a vast region of the Arctic. We further compare the observed temperature dependence of isoprene emissions with predictions from the Model of Emissions of Gases and Aerosols from Nature version 2.1 (MEGAN2.1), a widely used modeling framework for estimating ecosystem–atmosphere BVOC fluxes (Guenther et al., 2012).

## 2 Material and methods

### 2.1 Study site

**Figure 1.**
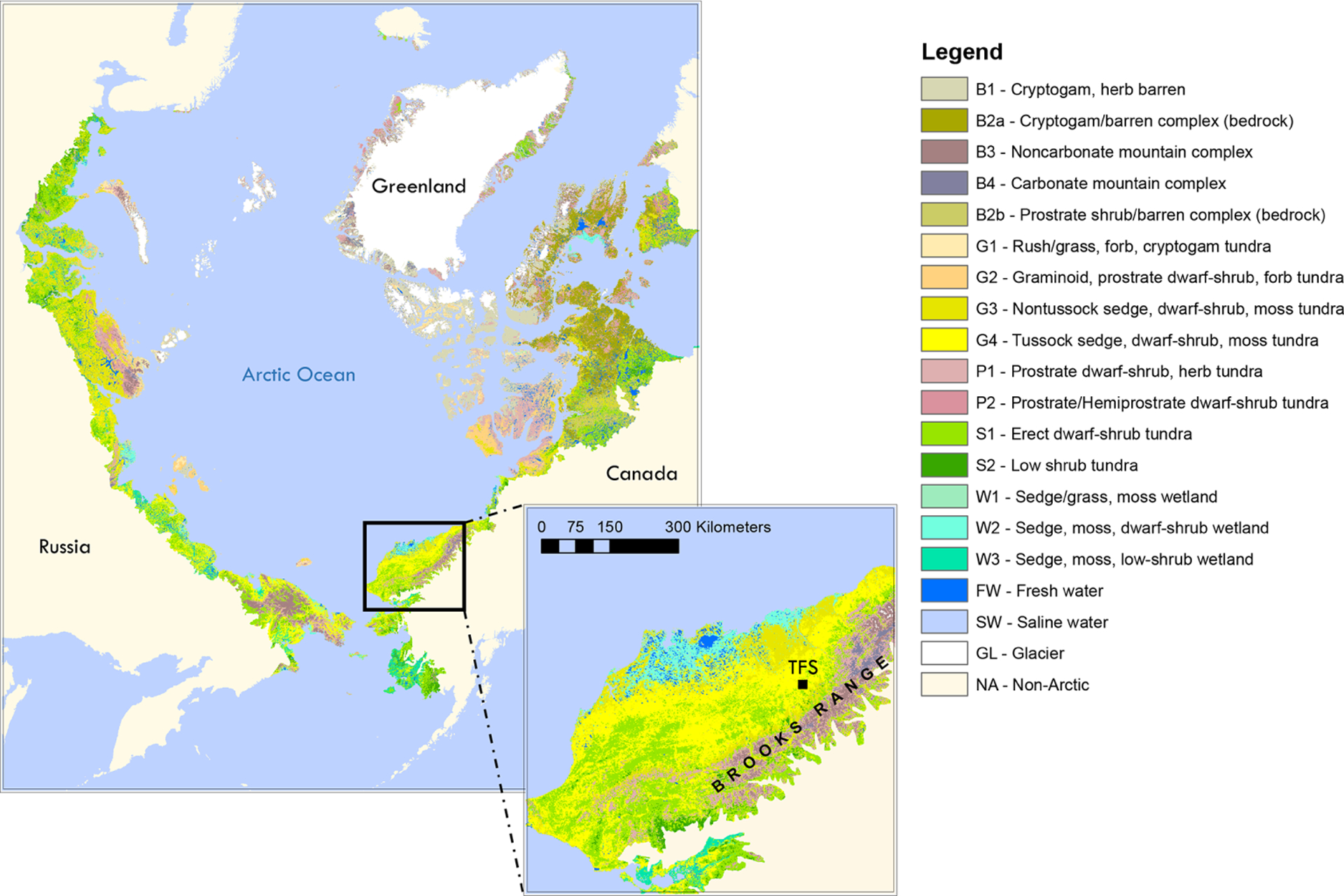
Location of Toolik Field Station (TFS) on the north flanks of the Brooks Range in northern Alaska along with arctic vegetation type. This figure was made using the raster version of the circumpolar Arctic vegetation map prepared by Raynolds et al. (2019), which is publicly available at https://www.geobotany.uaf.edu (last access: 19 May 2020).

This study was carried out at TFS, a Long-Term Ecological Research (LTER) site located in the tundra on the northern flank of the Brooks Range in northern Alaska (68°38′ N, 149°36′ W; see Fig. 1). Vegetation speciation and dynamics, and their changes over time, have been well documented at the site. *Betula* (birch) and *Salix* (willow) are the most common deciduous shrubs (Kade et al., 2012). Common plant species include *Betula nana* (dwarf birch), a major player in ongoing Arctic greening (Hollesen et al., 2015; Sistla et al., 2013), *Rhododendron tomentosum* (formerly *Ledum palustre*; Labrador tea), *Vaccinium vitis-idaea* (lowbush cranberry), *Eriophorum vaginatum* (cotton grass), *Sphagnum angustifolium* (peat moss), *Alectoria ochroleuca* (witches hair lichen), and many other perennial species of Carex, mosses, and lichens. Vegetation cover at this site is classified as tussock tundra (see Fig.1), which is the most common vegetation type in the northern foothills of the Brooks Range (Elmendorf et al., 2012; Kade et al., 2012; Shaver and Chapin, 1991; Survey, 2012; Walker et al., 1994).

Emission measurements and atmospheric sampling were conducted from a weatherproof instrument shelter located ~ 350 m to the west of TFS (see Fig. S1 in the Supplement). Winds at TFS are predominantly from the southerly and northerly sectors (Toolik Field Station Environmental Data Center, 2019), minimizing any influence from camp emissions at the site. Two field campaigns were carried out; the first was from mid-July to mid-August 2018, and the second was from mid-May to the end of June 2019. These two back-to-back campaigns cover the entire growing season (Sullivan et al., 2007), from the onset of snowmelt in mid-May to the first snowfall in mid-August.

### 2.2 Ambient online measurements of BVOCs and their oxidation products

#### 2.2.1 Gas chromatography and mass spectrometry with flame ionization detector (GC-MS/FID)

An automated gas chromatography and mass spectrometry with flame ionization detector (GC-MS/FID) system was deployed for continuous measurements of atmospheric BVOCs at ~ 2 h time resolution during the 2018 and 2019 field campaigns. In addition, the system was operated remotely following the 2018 campaign (through 15 September) in order to collect background values at the beginning of autumn. Air was pulled continuously from an inlet on a 4 m meteorological tower located approximately 30 m from the instrument shelter (Van Dam et al., 2013). Air passed through a sodium thiosulfate-coated O_3_ scrubber for selective O_3_ removal – to prevent sampling losses and artifacts for reactive BVOCs (Helmig, 1997; Pollmann et al., 2005) – and through a moisture trap to dry the air to a dew point of −45 °C.The moisture trap was a u-shaped Silcosteel^™^ tube (stainless steel treated) cooled using thermoelectric coolers. Analytes were concentrated on a Peltier-cooled (−40 °C) multistage microadsorbent trap (50 % Tenax-GR and 50 % Carboxen 1016). Analysis was accomplished by thermal desorption and injection for cryogen-free GC using a DB-1 column (60 m × 320 μm × 5 μm) and helium as a carrier gas. The oven temperature was set to 40 °C for 6 min, then increased to 260 °C at 20 °C min^−1^, and held isothermally at 260 °C for 13 min. The column flow was split between an FID and a MS for simultaneous quantification and identification. Blanks and calibration standards were regularly injected from a manifold. Isoprene (*m/z* 67 and 68), methacrolein (MACR) and methyl vinyl ketone (MVK) (*m/z* 41, 55, and 70), MT (*m/z* 68, 93, 121, and 136), and SQT (*m/z* 204, 91, 93, 119, and 69) were identified and quantified using the MS in selected ion-monitoring mode (SIM). The response to isoprene was calibrated using a primary gas standard supplied by the National Physical Laboratory (NPL), certified as containing 4.01 ± 0.09 parts per billion (ppb) of isoprene in a nitrogen matrix. The analytical uncertainty for isoprene was estimated at 16 %, based on the certified uncertainty of the standard and on the repeatability of the standard analysis throughout the campaigns. Instrument responses for MACR, MVK, *α*-pinene, and acetonitrile were calibrated with multicomponent standards containing 1007 ppb MACR, 971 ppb MVK, 967 ppb *α*-pinene, and 1016 ppb acetonitrile (Apel-Riemer Environmental Inc., Miami, FL, USA) dynamically diluted into a stream of ultra-zero-grade air to ~ 3 ppb. Quantification of other terpenoid compounds was based on GC peak area (FID response) plus relative response factors using the effective carbon number concept (Faiola et al., 2012; Scanlon and Willis, 1985). The limit of quantification (LOQ) was ~ 2 parts per trillion by volume – pptv (pmol mol^−1^ by volume). In order to monitor and correct for long-term trends in the detection system, including detector drift and decreasing performance of the adsorbent trap, we used peak areas for long-lived chlorofluorocarbons (CFCs) that were monitored in the air samples together with the BVOCs as an internal reference standard. The atmospheric trace gases, CCl_3_F (CFC-11) and CCl_2_FCCl_2_F_2_ (CFC-113), are ideal in this regard because they are ubiquitous in the atmosphere and exhibit little spatial and temporal variability (Karbiwnyk et al., 2003; Wang et al., 2000).

#### 2.2.2 Proton transfer reaction time-of-flight mass spectrometry (PTR-ToF-MS)

During the summer 2019 campaign, isoprene mixing ratios in ambient air were also measured by proton transfer reaction time-of-flight mass spectrometry (PTR-ToF-MS; model 4000, Ionicon Analytik GmbH, Innsbruck, Austria). The sample inlet was located on the 4 m meteorological tower, right next to the GC-MS/FID inlet. In brief, ambient air was continuously pulled through the PTR-ToF-MS drift tube, where volatile organic compounds (VOCs) with proton affinities higher than that of water (> 165.2 kcal mol^−1^) were ionized via a proton-transfer reaction with primary H_3_O^+^ ions, then subsequently separated and detected by a time-of-flight mass spectrometer (with a mass resolving power up to 4000). At TFS, the PTR-ToF-MS measured ions from *m/z* 17–400 every 2 min. Ambient air was drawn to the instrument at 10–15 L min^−1^ via ~ 30 m of 1*/*4″ (6.35 mm) outer diameter (OD) perfluoroalkoxy (PFA) tubing maintained at ~ 55 °C and then subsampled by the instrument through ~ 100 cm of 1*/*16″ (1.59 mm) OD polyetheretherketone (PEEK) tubing maintained at 60 °C. The residence time from the inlet on the 4 m meteorological tower to the drift tube was less than 5 s. Instrument backgrounds were quantified approximately every 5 h for 20 min during the campaign by measuring VOC-free air generated by passing ambient air through a heated catalytic converter (375 °C, platinum bead, 1 wt % Pt; Sigma Aldrich). Calibrations were typically performed every 4 d via dynamic dilution of certified gas standard mixtures containing 25 distinct VOCs, including isoprene (Apel-Riemer Environmental Inc., Miami, FL). Here, we report isoprene mixing ratios in order to intercompare them with GC-MS measurements; other species will be reported in future work. The measurement uncertainty for isoprene is ~ 25 %, which includes uncertainties in the gas standards, calibration method, and data processing.

#### 2.2.3 Instrument intercomparison

Figure S2 shows a comparison of the GC-MS and PTR-ToF-MS isoprene mixing ratios in ambient air. With a correlation coefficient of 0.93 and a linear regression slope of 0.7–1.0, the two measurements agreed within their combined measurement uncertainties, in line with earlier intercomparison studies (e.g., Dunne et al., 2018; de Gouw et al., 2003). Similarly, we found a correlation coefficient of 0.96 between GC-MS and PTR-ToF-MS MVK and MACR mixing ratios (not shown). The good agreement between these two independent techniques gives us confidence that the ambient air results presented here are robust.

### 2.3 Ambient air vertical profiles

Vertical isoprene mixing ratio profiles were obtained using a 12 ft. (3.66 m) diameter SkyDoc tethered balloon. A total of eight vertical profiles were performed at ~ 3 h intervals between 12:30 Alaska standard time (AST; hereafter all times are given in AST) on 15 June 2019 and 11:00 on 16 June 2019 in order to capture a full diurnal cycle (solar noon around 14:00). Sampling packages were connected to the tether line such that resulting sampling heights were ~ 30, ~ 100, ~ 170, and ~ 240 m above ground level (a.g.l.). One identical sampling package was deployed at the surface. Each sampling package contained an adsorbent cartridge for sample collection (see below) that was connected to a downstream battery-powered SKC pocket pump controlled using a mechanical relay, a programmable Arduino, and a real-time clock. Once the balloon reached its apex (~ 250–300 m a.g.l.), the five pumps were activated simultaneously, and samples were collected for 30 min to ensure that enough material was collected. It should be noted that changes in wind speed and turbulence during the 30 min sampling period often affected the shape of the tethered line and the sampling altitude, adding further uncertainty to the vertical profiles presented here. At the end of the 30 min sampling period, the balloon was brought back down. The adsorbent cartridges were prepared in-house, using glass tubing (89 mm long × 6.4 mm OD; 4.8 mm inner diameter – ID), and loaded with Tenax-GR and Carboxen 1016 adsorbents (270 mg of each), following established practice (Ortega and Helmig, 2008 and references therein). An inlet ozone scrubber was installed on each cartridge to prevent BVOC sampling losses. Field blanks were collected by opening a cartridge (with no pumped airflow) during each balloon flight. Following collection, adsorbent cartridges were sealed with Teflon-coated brass caps and stored in the dark at ~ 4 °C until the chemical analysis. Samples were analyzed at the University of Colorado Boulder, following the method described in Sect. S1 in the Supplement. Our previous intercomparison of this cartridge–GC-MS/FID method with independent and concurrent PTR-MS observations showed that the two measurements agree to within their combined uncertainties at ~ 25 % (Hu et al., 2015). Meteorological conditions were monitored and recorded during each balloon flight with a radiosonde (Met One Instruments, Inc., Grants Pass, OR, USA) attached to the tethered line just below the balloon.

### 2.4 BVOC emission rates

#### 2.4.1 Dynamic enclosure measurements

**Figure 2.**
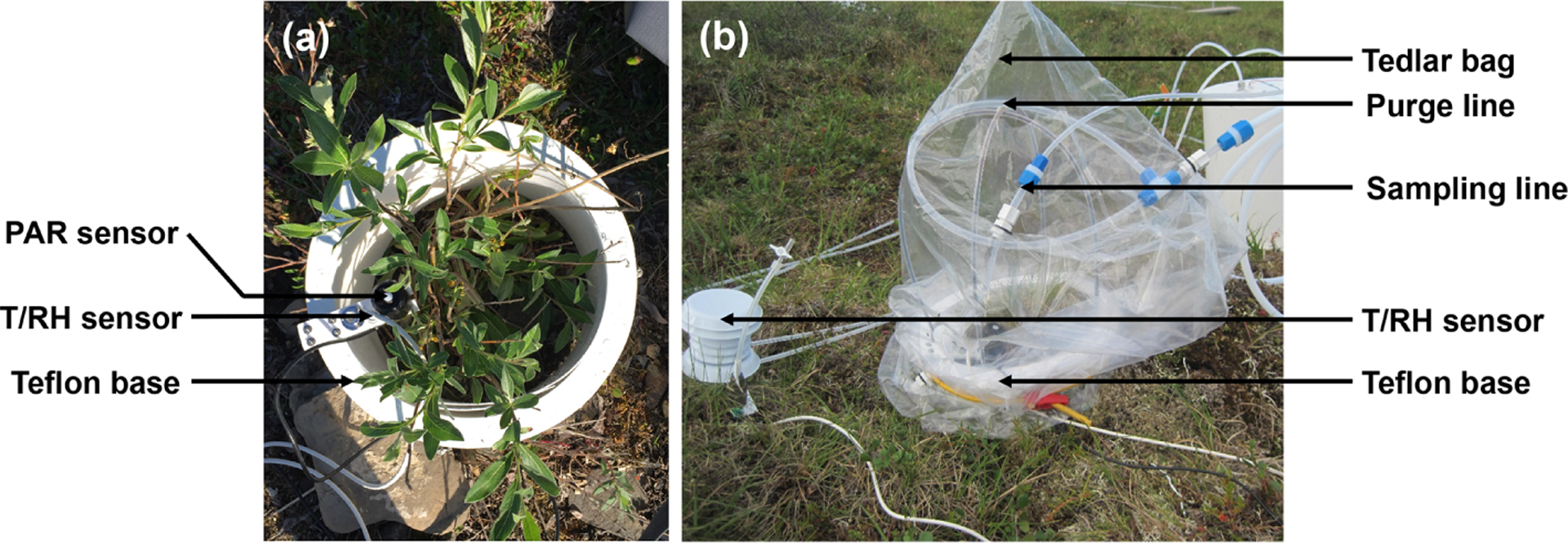
Photographs of a surface enclosure experiment setup at Toolik Field Station, Alaska. (**a**) The first step of the installation consisted of positioning the Teflon base around the vegetation of interest along with temperature (*T*), relative humidity (RH), and photosynthetically active radiation (PAR) sensors. (**b**) The second step consisted of positioning the Tedlar® bag around the base. The bag was connected to a purge-air and a sampling line. An additional *T/*RH sensor was also positioned outside the bag.

We used dynamic enclosure systems operated at low residence time to quantify vegetative BVOC emissions, following the procedure described by Ortega et al. (2008) and Ortega and Helmig (2008). Two types of enclosures were used, namely branch and surface chambers. For branch enclosures, a Tedlar® bag (Jensen Inert Products, Coral Springs, FL) was sealed around the trunk side of a branch. For surface enclosures, the bag was placed around a circular Teflon base (25 cm width × 16 cm height; see Fig. 2). For both the branch and surface enclosures, the bag was connected to a purge-air line and a sampling line and positioned around the vegetation, minimizing contact with foliage. While purging the enclosure (see Sect. 2.4.3), the vegetation was allowed to acclimate for 24 h before BVOC sampling began. Samples were collected from the enclosure air, concentrated onto solid-adsorbent cartridges (see Sect. 2.3) with an automated sampler, and analyzed in the laboratory at the University of Colorado Boulder following the campaign (see Sect. S1). Temperature and relative humidity were recorded inside and outside the enclosure (see Fig. 2; S-THB-M002 sensors, HOBO, Onset, Bourne, MA) with a data logger (H21-USB, HOBO, Onset, Bourne, MA). Additionally, photosynthetically active radiation (400–700 nm; S-LIA-M003; HOBO, Onset, Bourne, MA) was measured inside the enclosure. Once installed, enclosures were operated for 2–10 d. The tundra vegetation around TFS is heterogeneous, but the most dominant species (except *Rubus chamaemorus*) were sampled. Table 1 presents the median relative percent cover of plant species in LTER experimental control plots at TFS (Gough, 2019) and indicates whether plant species were present in surface or bag enclosures. The complete list of species sampled and pictures of the enclosures are shown in Figs. S3–S15; the two sampling sectors are highlighted in Fig. S1. Surface enclosures were divided into three vegetation types, namely *Salix* spp. (high isoprene emitter), *Betula* spp. (e.g., *Betula nana* dominance), and miscellaneous (a mix of different species, including lichens and mosses).

#### 2.4.2 Emission rates

The emission rate (ER in μgC m^−2^ h^−1^) for surface enclosures was calculated as follows:

(1) $${\text{ER}}_{\text{surface}}=\frac{\left(C_{\text{out}}-C_{\text{in}}\right)Q}{S},$$

where *C*_in_ and *C*_out_ are the inlet and outlet analyte concentrations (in μgC L^−1^), *Q* is the purge-air flow rate (in L h^−1^), and *S* is the surface area of the enclosure (in m^2^).

The ER for branch enclosures (in μgC g^−1^ h^−1^) was calculated as follows:

(2) $${\text{ER}}_{\text{branch}}=\frac{\left(C_{\text{out}}-C_{\text{in}}\right)Q}{m_{\text{dry}}},$$

where *m*_dry_ is the dried mass (in grams) of leaves enclosed, determined by drying the leaves – harvested after the experiment – at 60–70 °C until a consistent weight was achieved (Ortega and Helmig, 2008).

ERs were standardized to 30 °C and to a PAR level of 1000 μmol m^−2^ s^−1^ using the algorithms described in Guenther et al. (1993, 1995).

#### 2.4.3 Enclosure purge air

Purge air was provided by an upstream, high-capacity oil-free pump providing positive pressure to the enclosure and equipped with an in-line O_3_ scrubber to avoid a loss in reactive BVOCs from reaction with O_3_ in the enclosure air and during sampling (Helmig, 1997; Pollmann et al., 2005). The purge-air flow was set to 25 L min^−1^ and regularly checked using a volumetric flow meter (DryCal Defender, Mesa Labs Bios, Butler, NJ). Excess air escaped from the open end (tied around the Teflon base) while the sample air flow was pulled into the sampling line (see below).

#### 2.4.4 Sample collection

A continuous airflow of 400–500 mL min^−1^ was drawn from the enclosure through the sampling line. A fraction of this flow was periodically collected at 265–275 mL min^−1^ on adsorbent cartridges (see Sect. 2.3) using a 10-cartridge autosampler (Helmig et al., 2004). During sampling, cartridges were kept at 40 °C, i.e., above ambient temperature, to prevent water accumulation on the adsorbent bed (Karbiwnyk et al., 2002). Samples were periodically collected in series to verify lack of analyte breakthrough. Time-integrated samples were collected for 120 min every 2 h to establish diurnal cycles of BVOC emission. Upon collection, samples were stored in the dark at ~ 4 °C until the chemical analysis at the University of Colorado Boulder.

#### 2.4.5 Internal standards

In order to identify potential BVOC losses during transport, storage, and chemical analysis, 255 of the employed cartridges were preloaded with a four-compound standard mixture prior to the field campaigns. These internal standard compounds (toluene, 1,2,3-trimethylbenzene, 1,2,3,4-tetrahydronaphthalene, and 1,3,5-triisopropylbenzene) were carefully chosen to span a wide range of volatility (C_7_–C_15_) and to not interfere (i.e., coelute) with the targeted BVOCs. The recovery of these four compounds was assessed at the end of the campaign, following the analytical procedure described in Sect. S1. Recovery rates were 101.8 ± 13.5 % (toluene), 95.2 ± 20.1 % (1,2,3-trimethylbenzene), 95.6 ± 26.6 % (1,2,3,4-tetrahydronaphthalene), and 100.9 ± 18.7 % (1,3,5-triisopropylbenzene). These results indicate that, overall, BVOC losses during transport, storage, and chemical analysis were negligible. Ortega et al. (2008) previously evaluated the systematic losses of analytes to enclosure systems similar to those used here. The same four-component standard was introduced into the purge-air flow of the enclosures to quantify losses as a function of volatility. That work found median losses of MT and SQT of the order of 20 %–30 %. The emission rates presented here are therefore possibly biased to be lower by a similar amount.

### 2.5 Peak fitting algorithm

The analysis of ambient air and enclosure chromatograms was performed using the TERN (Thermal desorption aerosol gas chromatography ExploreR and iNtegration package) peak fitting tool implemented in Igor Pro and available online at https://sites.google.com/site/terninigor/(last access: 19 January 2020; Isaacman-VanWertz et al., 2017).

### 2.6 Ancillary parameters

#### Meteorological parameters.

A suite of meteorological instruments was deployed on the 4 m tower. Wind speed and direction were measured at ~ 4 m a.g.l. with a 034B-L sensor (Met One Instruments, Inc., Grants Pass, OR, USA). As described by Van Dam et al. (2013), temperature was measured at three different heights using resistance temperature detector (RTD) temperature probes (model 41342, R. M. Young Company, Traverse City, MI) housed in aspirated radiation shields (model 43502; R. M. Young Company, Traverse City, MI). Regular same-height intercomparisons were conducted to test for instrumental offsets. Incoming and reflected solar radiation were recorded with LI200X pyranometers (Campbell Scientific).

In addition, historical (1988–2019) meteorological data recorded by the TFS Environmental Data Center are available at: https://toolik.alaska.edu/edc/abiotic_monitoring/data_query.php (last access: 15 April 2020).

#### Particle measurements.

A Met One Instruments, Inc., Model 212–2 eight-channel (0.3 to 10 μm) particle profiler was operated continuously on the roof of the weatherproof instrument shelter. This instrument uses a laser-diode-based optical sensor and light-scatter technology to detect, size, and count particles (http://mail.metone.com/particulate-Aero212.htm, last access: 12 February 2020).

#### Nitrogen oxides.

Nitrogen oxides (NO_*x*_) were measured with a custom-built, high sensitivity (~ 5 pptv detection limit) single-channel chemiluminescence analyzer (Fontijn et al., 1970). The instrument monitors nitric oxide (NO) and nitrogen dioxide (NO_2_) in ambient air using a photolytic converter. Automated switching valves alternated between the NO and NO_2_ mode every 30 min. Calibration was accomplished by dynamic dilution of a 1.5 parts per million (ppm) compressed NO gas standard (Scott-Marrin, Inc., Riverside, CA).

### 2.7 Theoretical response of isoprene emissions to temperature in MEGAN2.1

We applied our isoprene emission measurements to evaluate the temperature response algorithms embedded in MEGAN2.1 (Guenther et al., 2012). Theoretical isoprene emission rates (*F*_T_) were calculated for TFS as follows:

(3) $$F_{\text{T}}=C_{\text{CE}}\gamma_{\text{T}}\sum_{j}\kappa_{j}\varepsilon_{j},$$

where *C*_CE_ is the canopy environment coefficient (assigned a value that results in *γ*_T_ 1 under standard conditions), and ε_*j*_ is the emission factor under standard conditions for vegetation type *j* with fractional grid box areal coverage *κ*_*j*_. We used $\sum_{j}\kappa_{j}\varepsilon_{j}=2766\;\mu \text{g}\;{\text{m}}^{-2}\;{\text{h}}^{-1}$ at TFS, based on the high resolution (1 km) global emission factor input file available at https://bai.ess.uci.edu/megan/data-and-code/megan21 (last access: 21 May 2020). The temperature activity factor (*γ*_T_) was calculated as the following:

(4) $$\gamma_{\text{T}}=E_{\text{opt}}\times \frac{200\;e^{95x}}{200-95\times \left(1-e^{200\;x}\right)},$$

with

(5) $$x=\frac{\frac{1}{T_{\text{opt}}}-\frac{1}{T}}{0.00831}$$

(6) $$E_{\text{opt}}=2\times e^{0.08\left(T_{10}-297\right)}$$

(7) $$T_{\text{opt}}=313+0.6\left(T_{10}-297\right),$$

where *T* is the enclosure ambient air temperature, and *T*_10_ is the average enclosure air temperature over the past 10 d.

## 3 Results and discussion

### 3.1 Ambient air mixing ratios

#### 3.1.1 Isoprene and oxidation products

**Figure 3.**
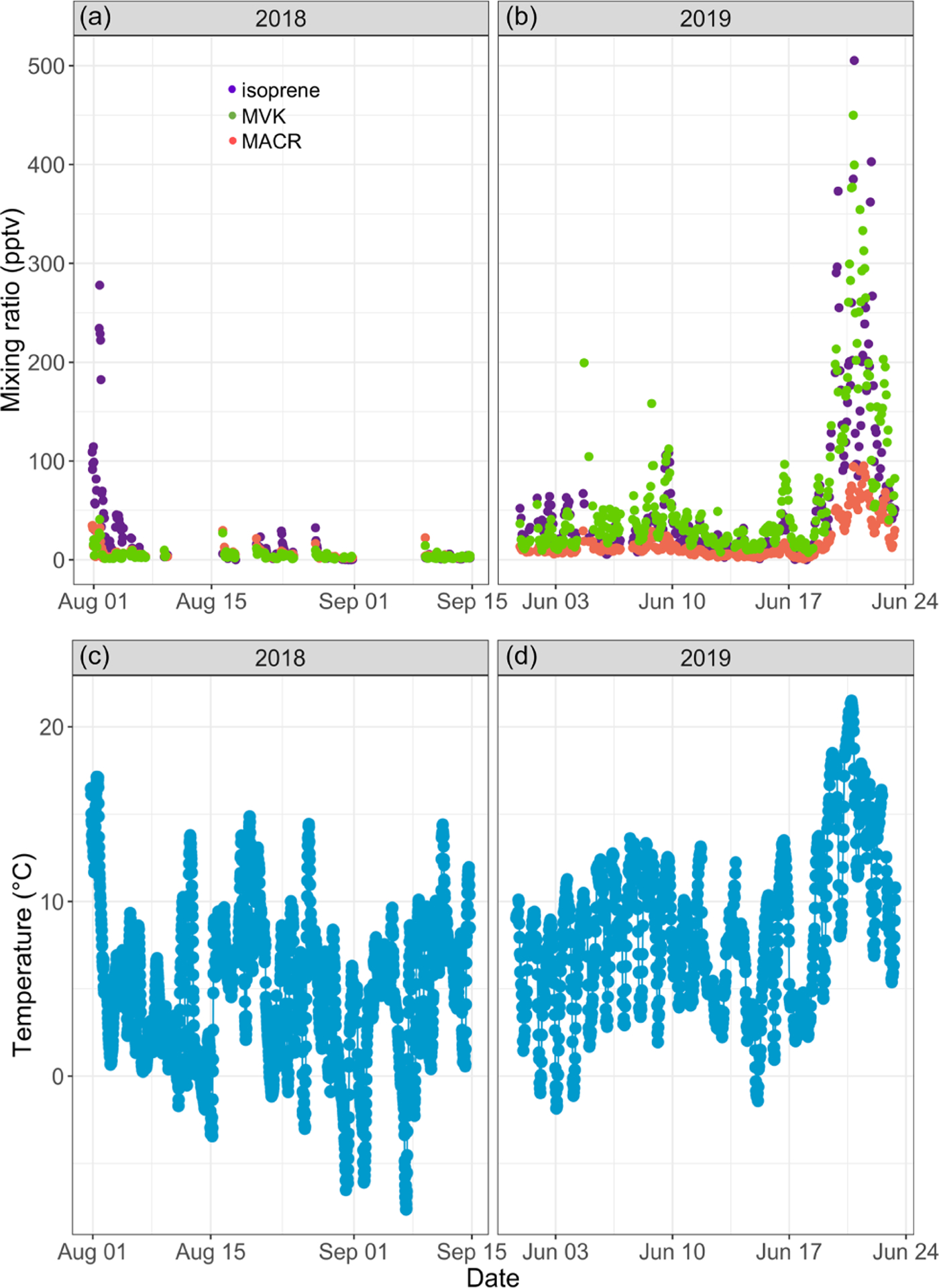
Time series of isoprene (purple), methyl vinyl ketone (MVK; green), and methacrolein (MACR; salmon) mixing ratios (in parts per trillion by volume – pptv) in ambient air at Toolik Field station (**a, b**) and of 30 min averaged ambient temperature (in degrees Celsius) at 4 m above ground level (**c, d**).

Figure 3a and b show the time series of isoprene mixing ratios in ambient air recorded over the course of this study at TFS with the GC system. Mixing ratios were highly variable and ranged from below the quantification limit to 505 pptv (mean of 36.1 pptv). The PTR-ToF-MS gave similar results (see Fig. S16a). These mixing ratios fall within the range of values reported in the Eurasian taiga (e.g., Hakola et al., 2000, 2003; Lappalainen et al., 2009). For example, Hakola et al. (2003) reported a maximum monthly mean mixing ratio of 98 pptv (in July) in central Finland, while Hakola et al. (2000) observed mixing ratios ranging from a few pptv to ~ 600 pptv in eastern Finland. In general, however, BVOC emissions in the Eurasian taiga are relatively low compared to forest ecosystems in warmer climates and are dominated by monoterpenes (Rinne et al., 2009).

**Figure 4.**
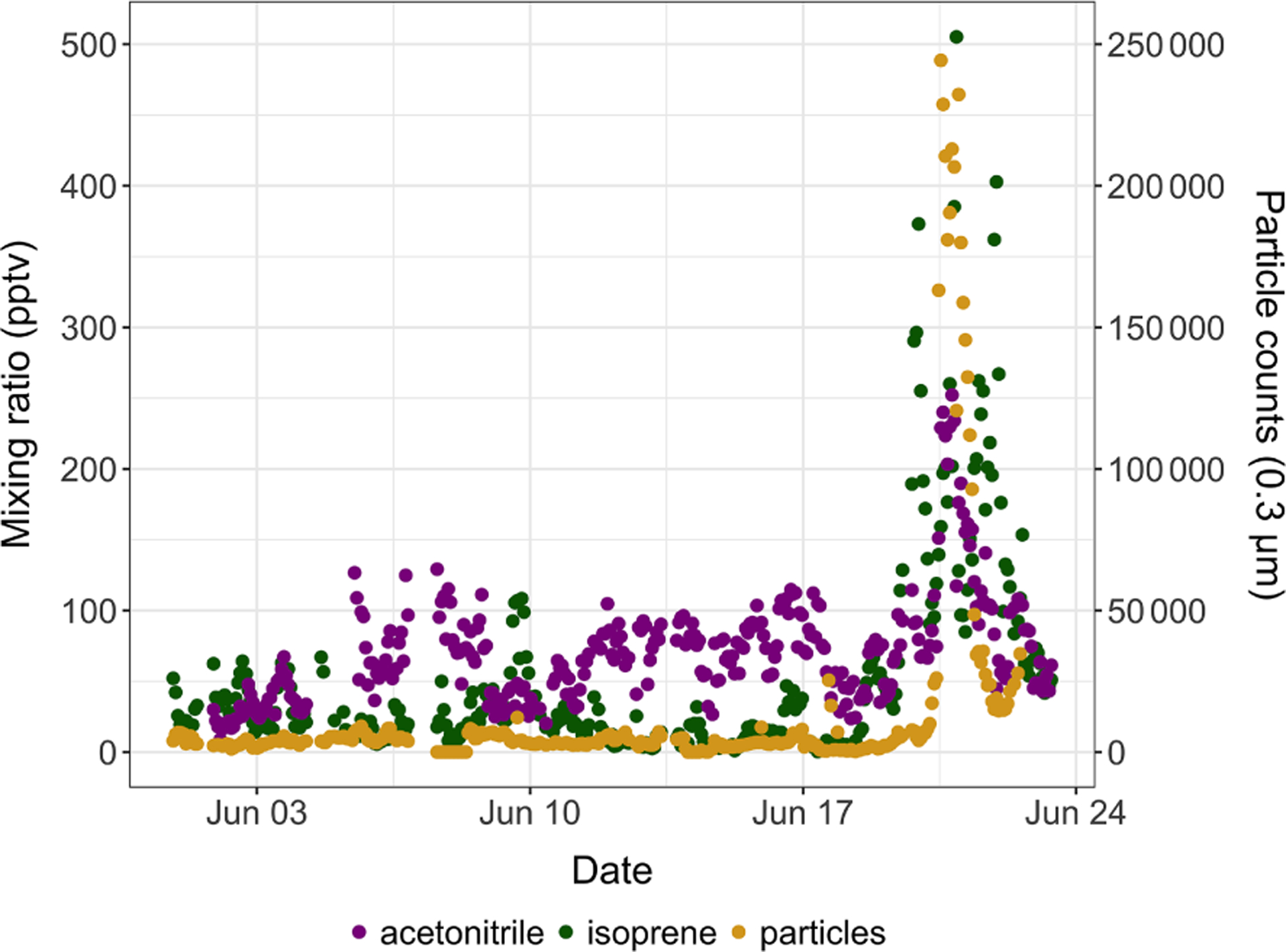
Time series of isoprene (green) and acetonitrile (purple) mixing ratios (in pptv) and of 0.3 μm particle counts (yellow) in ambient air at Toolik Field station in June 2019.

Isoprene mixing ratios peaked on 1 August 2018 around 16:00 and on 20 June 2019 around 22:00, respectively. These two peaks occurred 3–5 h after the daily maximum ambient temperature was reached (17.8 °C in 2019 and 21.8 °C in 2019; see Fig. 3). The isoprene peak on 20 June 2019 was concomitant with enhanced acetonitrile mixing ratios and particle counts (see Fig. 4), reflecting unusually hazy conditions that day at TFS. We attribute the particle and acetonitrile enhancements to intense wildfires occurring across the Arctic Circle at that time, with most of them being in southern Alaska and Siberia (Earth Observatory, 2019). Acetonitrile increased by a factor of 4 during this event, compared to a factor of 21 increase for isoprene. The higher emission factor for acetonitrile vs. isoprene from biomass burning in boreal forests (Akagi et al., 2011) and the relatively short lifetime of isoprene (Atkinson, 2000) indicate that the observed isoprene enhancement was due to fresh local biogenic emissions rather than transported wildfire emissions.

Over the course of this study, we recorded MACR and MVK mixing ratios, respectively, ranging from below the quantification limit to 95 pptv (12.4 ± 16.1 pptv; mean ± standard deviation) and from below the quantification limit to 450 pptv (43.1 ± 66.7 pptv; see Fig. 3a, b). The PTR-ToF-MS gave similar results (see Fig. S16b). Median NO and NO_2_ mixing ratios of 21 and 74 pptv, respectively, during the 2019 campaign (not shown) suggest a low NO_*x*_ environment, in line with previous studies at several Arctic locations (Bakwin et al., 1992; Honrath and Jaffe, 1992). Under such conditions, MACR and MVK mixing ratios should be used as upper estimates as it has been noted that some low NO_*x*_ isoprene oxidation products (isoprene hydroxyhydroperoxides) can undergo rearrangement in GC and PTR-MS instruments and be misidentified as MACR and MVK (Rivera-Rios et al., 2014). We found a high correlation between MACR and MVK (*R*^2^ = 0.95, *p <* 0.01) and between these two compounds and isoprene (*R*^2^ ~ 0.80, *p <* 0.01). Increases in MACR and MVK mixing ratios above the background were mostly concomitant with isoprene increases, suggesting that atmospheric or within-plant oxidation of isoprene was their main source (Biesenthal et al., 1997; Hakola et al., 2003; Jardine et al., 2012). The mean ratio of MVK to MACR was 2.7, within the range reported by earlier studies (e.g., Apel et al., 2002; Biesenthal and Shepson, 1997; Hakola et al., 2003; Helmig et al., 1998), and no clear diurnal cycle in the ratio was found. This record of ambient air isoprene, MACR, and MVK mixing ratios is, to the best of our knowledge, the first in an Arctic tundra environment. The combined measurement of isoprene and its oxidation products provides a new set of observations to further constrain isoprene chemistry under low NO_*x*_ conditions in atmospheric models (e.g., Bates and Jacob, 2019).

#### 3.1.2 Isoprene vertical profiles

**Figure 5.**
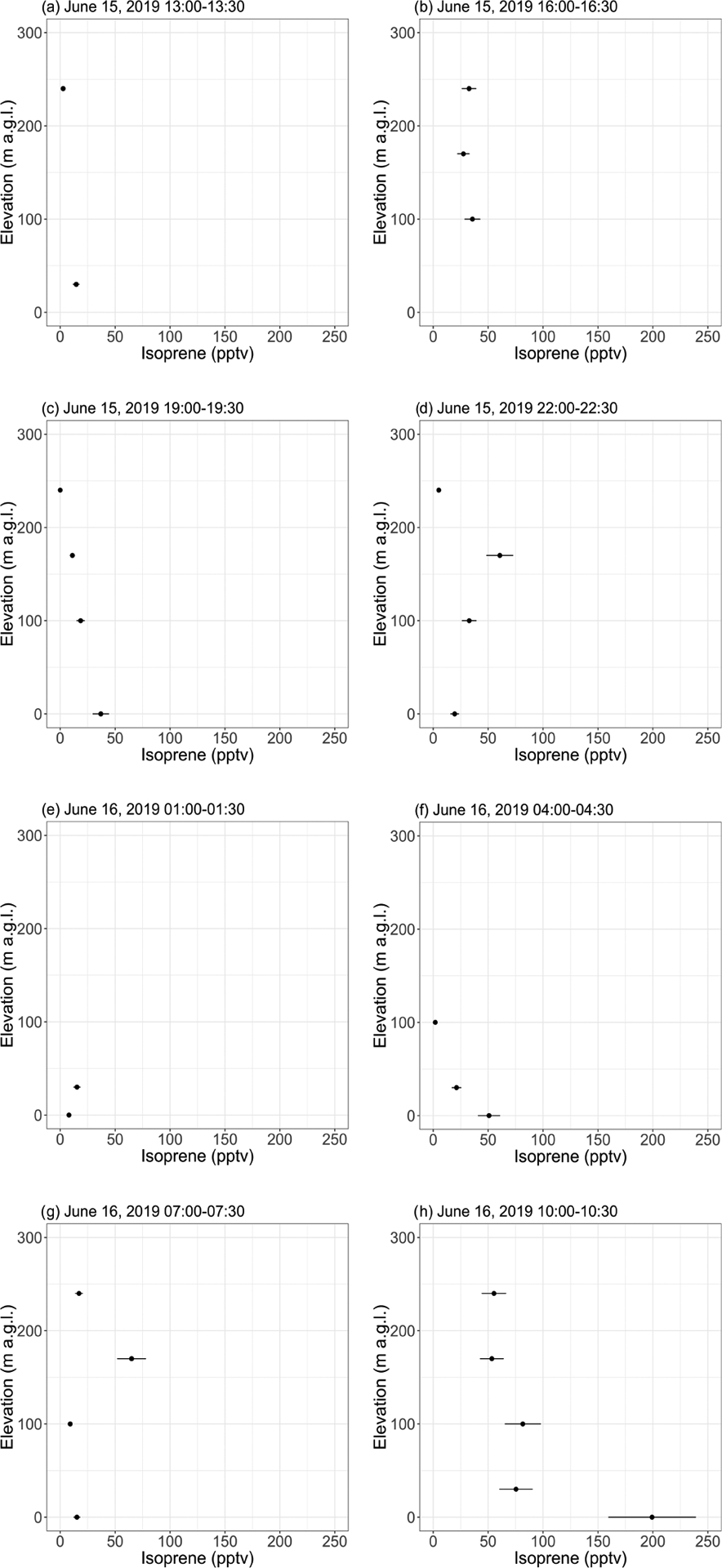
Vertical profiles of isoprene mixing ratios as inferred from 30 min samples collected with a tethered balloon. The error bars show the analytical uncertainty for isoprene (20 %). Samples with an isoprene mixing ratio lower than blanks were discarded. Times are given as Alaska standard time (UTC−9).

Figure 5 shows vertical profiles (0 to ~ 250 m a.g.l.) of isoprene mixing ratios derived from the 30 min tethered balloon samples collected on 15 and 16 June 2019. Temperature profiles (see Fig. S17) indicate that most of the flights were performed in a convective boundary layer (Holton and Hakim, 2013). A nocturnal boundary layer was, however, observed in the first ~ 50 m from ~ 02:00 to 04:30 (see Fig. S17e–f), with temperature increasing with elevation.

Except during the last flight, isoprene mixing ratios were in the range of background levels (~ 0–50 pptv) reported with the GC-MS (see Sect. 3.1.1). Samples collected from 10:00–10:30 on 16 June (see Fig. 5h) showed a pronounced gradient, with 200 pptv at ground level and decreasing mixing ratios with elevation. This maximum at ground level is expected for a VOC with a surface source (Helmig et al., 1998), while the 200 pptv mixing ratio can likely be attributed to a temperature-driven increase in isoprene emissions by the surrounding vegetation. Indeed, the ambient temperature at ground level was higher during that flight than during the previous ones (see Fig. S17h). The diurnal cycles of isoprene emissions and temperature are further discussed in Sect. 3.2.2. Interestingly, the GC-MS and the PTR-ToF-MS did not capture this 200 pptv maximum (see Figs. 3 and S16), which may be because the balloon flights were performed at a different location (near sampling sector B; see Fig. S1) surrounded by a higher fraction of isoprene-emitting shrubs (willow).

Samples collected on 16 June 2019 from 04:00 to 04:30 (see Fig. 5f) show decreasing isoprene mixing ratios with increasing elevation, suggesting higher levels (25–50 pptv) in the nocturnal boundary layer than above. This result suggests continuing isoprene emissions by the surrounding vegetation under low PAR conditions. This is further discussed in Sect. 3.2.2.

#### 3.1.3 Monoterpenes and sesquiterpenes

MT mixing ratios ranged from 3 to 537 pptv (14 ± 18 pptv; median ± standard deviation) during the 2019 campaign, according to the PTR-ToF-MS measurements. Using the GC-MS/FID, we were able to detect and quantify the following MT in ambient air: *α*-pinene, camphene, sabinene, p-cymene, and limonene. Mean mixing ratios are reported in Table 2 (for values lower than the LOQ, mixing ratios equal to half of the LOQ are used). These compounds have been previously identified as emissions of the widespread circumpolar dwarf birch *Betula nana* (Li et al., 2019; Vedel-Petersen et al., 2015) and other high Arctic vegetation (Schollert et al., 2014). The quantification frequency of camphene, sabinene, p-cymene, and limonene was low (see Table 2), and MT mixing ratios in ambient air were dominated by *α*-pinene. Several prior studies performed at boreal sites have similarly identified *α*-pinene as the most abundant monoterpene throughout the growing season (e.g., Hakola et al., 2000; Lindfors et al., 2000; Spirig et al., 2004; Tarvainen et al., 2007). We did not detect any sesquiterpene in ambient air above the 2 pptv instrumental LOQ.

Overall, isoprene and *α*-pinene dominated the ambient air BVOC profile at TFS, respectively constituting ~ 72 % and ~ 24 % of total BVOCs quantified in ambient air (on a mixing-ratio basis).

### 3.2 Emission rates

#### 3.2.1 Branch enclosures

A branch enclosure experiment was performed from 27 July to 2 August 2018 on *Salix glauca* to investigate BVOC emission rates per dry weight plant biomass (see Fig. S5). Isoprene emission rates ranged from < 0.01 to 11 μgC g^−1^ h^−1^ (with a mean enclosure temperature of 16.5 °C and mean PAR of 880 μmol m^−2^ s^−1^), in line with nonnormalized emission rates reported at Kobbefjord, Greenland, by Kramshøj et al. (2016; Table 5 in their Supplement) for the same species under slightly different environmental conditions (mean temperature of 24.6 °C and mean PAR of 1052 μmol m^−2^ s^−1^). Once standardized to 30 °C and 1000 μmol m^−2^ s^−1^, our emission rates averaged 5 μgC g^−1^ h^−1^, in good agreement with standardized emissions reported at Kobbefjord (mean of 7 μgC g^−1^ h^−1^) by Vedel-Petersen et al. (2015). The quantified MTs had emissions averaging 2 orders of magnitude lower than those of isoprene (0.01 vs. 1 μgC g^−1^ h^−1^). Emission rates for the sum of *α*-pinene, *β*-pinene, limonene, camphene, and 1,8-cineole ranged from < 0.01 to 0.06 μgC g^−1^ h^−1^. These results are again in good agreement with those reported for the same species at Kobbefjord (~ 0.01 μgC g^−1^ h^−1^) by Kramshøj et al. (2016; Table 5 in their Supplement).

#### 3.2.2 Surface emission rates

**Figure 6.**
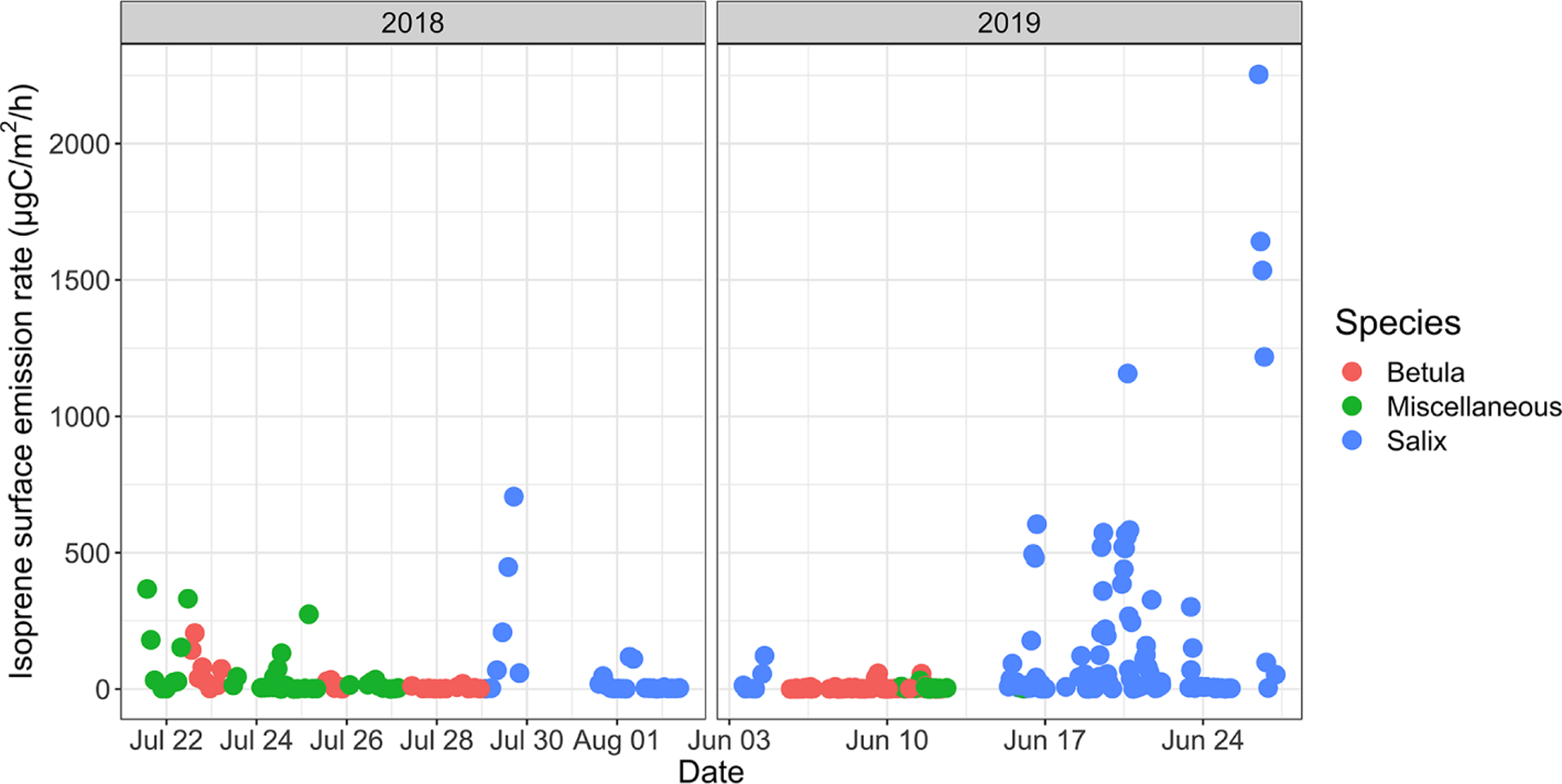
Time series of isoprene surface emission rates (in μgC m^−2^ h^−1^) for different vegetation types. Miscellaneous refers to a mix of different species, including lichens and moss tundra.

The isoprene surface emission rate, as inferred from surface enclosures, was highly variable and ranged from 0.2 to ~ 2250 μgC m^−2^ h^−1^ (see Fig. 6). The 2250 μgC m^−2^ h^−1^ maximum, reached on 26 June 2019, with an enclosure temperature of 32 °C, is higher than maximum values reported at TFS by Potosnak et al. (2013) (1200 μgC m^−2^ h^−1^ at an air temperature of 22 °C). It should be noted that these maximum values were observed at different ambient temperatures; we further investigate the temperature dependency of isoprene emissions in Sect. 3.3. Elevated surface emission rates (i.e., > 500 μgC m^−2^ h^−1^) were all observed while sampling enclosures dominated by *Salix* spp. At TFS, the overall 24 h mean isoprene emission rate amounted to 85 μgC m^−2^ h^−1^, while the daytime (10:00–20:00) and midday (11:00–14:00) means were 140 and 213 μgC m^−2^ h^−1^, respectively. To put this in perspective, the average isoprene surface emission rate standardized to 30 °C and 1000 μmol m^−2^ s^−1^ (~ 300 μgC m^−2^ h^−1^) was an order of magnitude lower than emission rates reported for warmer midlatitude or tropical forests. For example, average midday fluxes of 3000 μgC m^−2^ h^−1^ were reported in a northern hardwood forest in Michigan (Pressley et al., 2005), while several reports of isoprene emissions from tropical ecosystems give daily estimates of 2500–3000 μgC m^−2^ h^−1^ (Helmig et al., 1998; Karl et al., 2004; Rinne et al., 2002).

**Figure 7.**
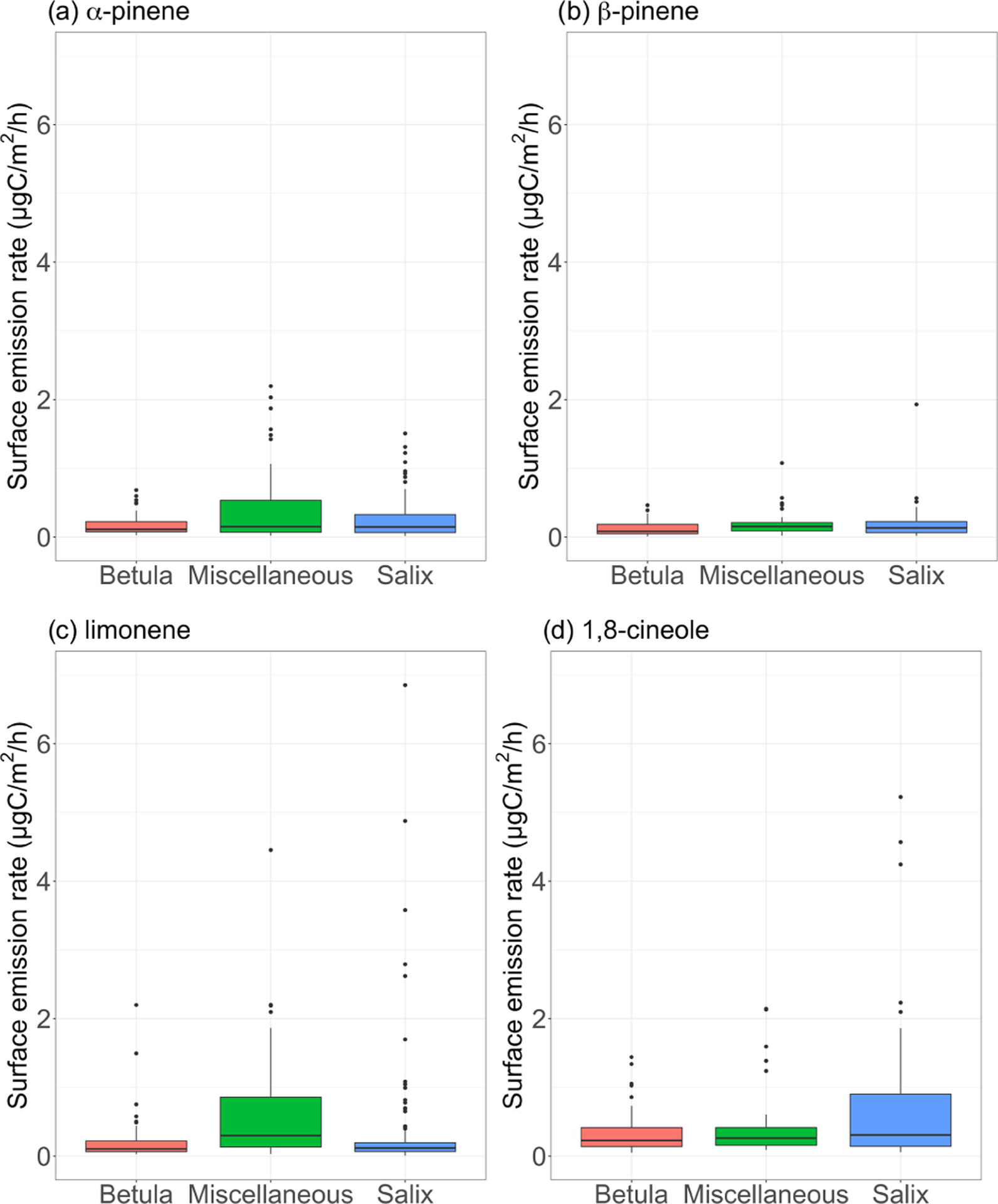
Surface emission rates of various monoterpenes (in μgC m^−2^ h^−1^) for different vegetation types. The lower and upper hinges correspond to the first and third quartiles. The upper (lower) whisker extends from the hinge to the largest (smallest) value no further than 1.5 × IQR from the hinge, where IQR is the interquartile range (i.e., the distance between the first and third quartiles). The notches extend $1.58\times \text{IQR}/\sqrt{n}$ and give a ~95 % confidence interval for medians. Miscellaneous refers to a mix of different species, including lichens and moss tundra.

Figure 7 shows the measured surface emission rates for *α*-pinene, *β*-pinene, limonene, and 1,8-cineole. While p-cymene, sabinene, 3-carene, and isocaryophyllene (SQT) were detected in some of the surface enclosure samples, we focus the discussion on the most frequently quantified compounds. It is worth noting that the most frequently observed compounds in enclosure samples are among the most frequently seen MT in ambient air (see Sect. 3.1.3). Regardless of the species, emission rates remained, on average, below 1 μgC m^−2^ h^−1^ over the course of the study (see Table 3). These results are at the low end of emission rates reported for four vegetation types in high Arctic Greenland (Schollert et al., 2014) but are in line with results reported at Kobbefjord, Greenland, by Kramshøj et al. (2016; Table 4 in their Supplement).

**Figure 8.**
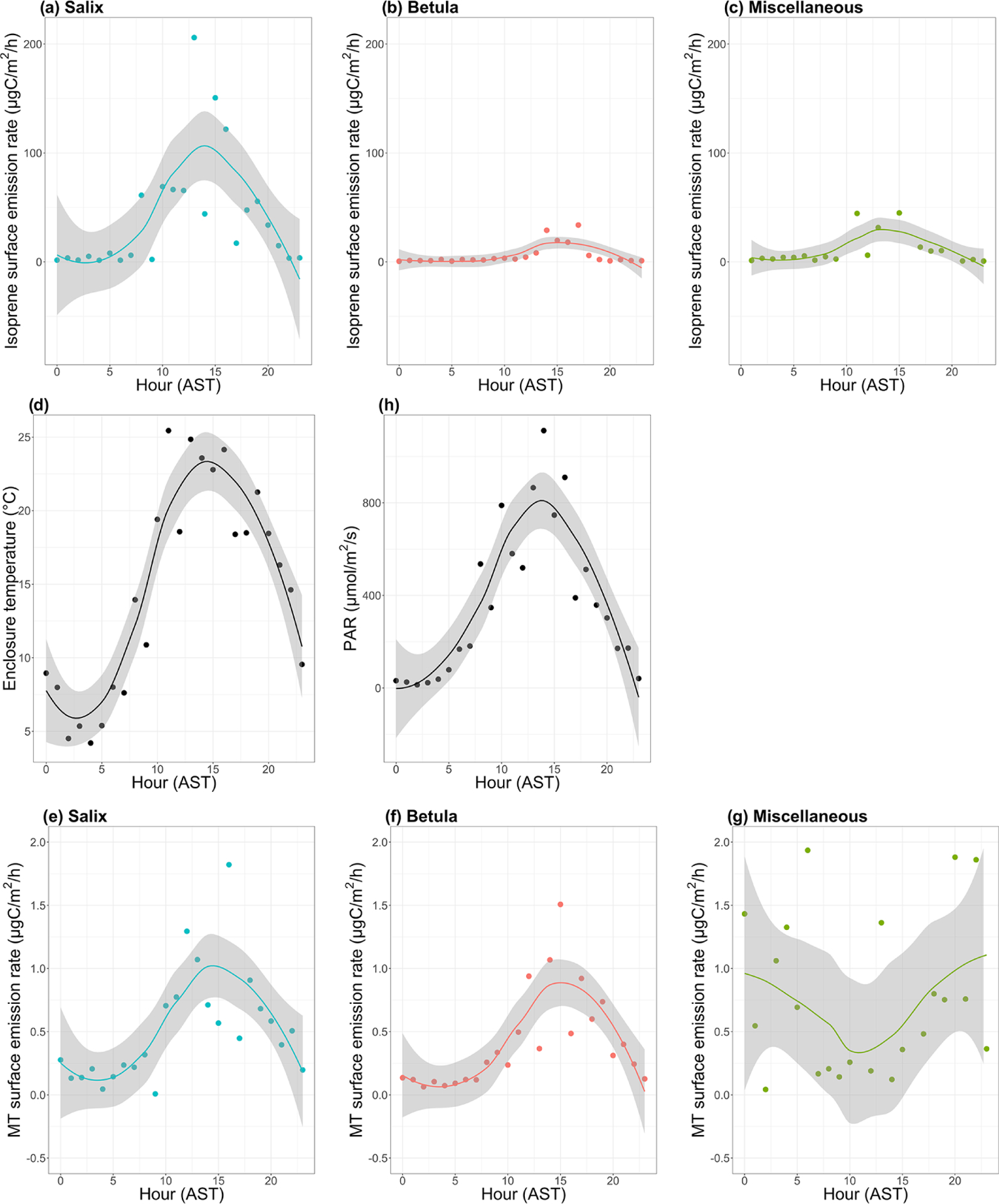
Mean diurnal cycle of isoprene (**a–c**) and monoterpenes (MT; **e–g**) surface emission rates (in μgC m^−2^ h^−1^; note the difference scale on the *y* axis), (**d**) enclosure temperature (in degrees Celsius), and (**h**) enclosure photosynthetically active radiation (PAR in μmol m^−2^ s^−1^). The dots represent the hourly means. The line is the smoothed conditional mean while the gray shaded region indicates the 95 % confidence interval. Hours are in Alaska standard time (UTC 9) and correspond to the end of the 2 h sampling period for isoprene and MT emission rates. MT corresponds here to the sum of *α*-pinene, *β*-pinene, limonene, and 1,8-cineole. Miscellaneous refers to a mix of different species, including lichens and moss tundra.

Figure 8a–c show the mean diurnal cycle (over the two campaigns) of isoprene surface emission rates for different vegetation types (see Fig. S3–S15 for nomenclature). The two field campaigns were carried out during the midnight sun period, which could possibly sustain BVOC emissions during nighttime. It should, however, be noted that low sun angles translate to very low PAR, and a typical diurnal pattern is observed in summer at TFS despite 24 h of light (see Fig. 8h). Regardless of the vegetation type, isoprene emission rates exhibited a significant diurnal cycle with an early afternoon maximum, in line with the mean diurnal cycle of the enclosure temperature and PAR. These results are in line with the well-established diurnal variation in BVOC emissions in environments ranging from Mediterranean to boreal forests (e.g., Fares et al., 2013; Liu et al., 2004; Ruuskanen et al., 2005; Zini et al., 2001) and with the correlation between isoprene ambient air mixing ratios and temperature at TFS (see Sect. 3.1). Despite the relatively low MT emission rates, a significant diurnal cycle was also observed, with peak total MT emissions of ~ 1 μgC m^−2^ h^−1^ during early afternoon for both *Salix* spp. and *Betula* spp. (Fig. 8e–f). A summary of emission rates per vegetation type and time of day is given in Table 3. As can be seen in Table 3 and Fig. 8, PAR and BVOC emissions significantly decreased at night but were still detectable. These sustained BVOC emissions during nighttime confirm observations by Lindwall et al. (2015) during a 24 h experiment with five different Arctic vegetation communities and explain the higher isoprene levels observed in rather than above the nocturnal boundary layer during the diurnal balloon experiment (see Sect. 3.1.2).

The ratio of total MT (given by the sum of *α*-pinene, *β*-pinene, limonene, and 1,8-cineole) emissions to isoprene emissions was an order of magnitude higher for *Betula* spp. (0.22) than for *Salix* spp. (0.03). This result, driven by the relatively lower isoprene emissions of *Betula* spp., is in line with earlier studies, suggesting similar emission characteristics for Arctic plants (e.g., Kramshøj et al., 2016; Vedel-Petersen et al., 2015).

## 4 Insights into future changes

### 4.1 Response of isoprene emissions to temperature

The Arctic has warmed significantly during the last three decades and temperatures are projected to increase by an additional 5–13 °C by the end of the century (Overland et al., 2014). Heat wave frequency is also increasing in the terrestrial Arctic (Dobricic et al., 2020). For example, western Siberia experienced an unusually warm May in 2020, with temperatures of 20–25 °C (Freedman and Cappucci, 2020). In that context, numerous studies have pointed out the likelihood of increased BVOC emissions due to Arctic warming and associated vegetation and land cover change (Faubert et al., 2010; Potosnak et al., 2013; Rinnan et al., 2011; Tiiva et al., 2008).

Over the course of the two field campaigns at TFS, BVOC surface emission rates were measured over a large span of enclosure temperatures (2–41 °C). While isoprene and MT emissions respond to leaf temperature (Guenther et al., 1993), air temperature was used here instead of leaf temperature, which has been assumed before in the literature for high-latitude ecosystems (e.g., Olofsson et al., 2005; Potosnak et al., 2013). Several studies have, however, suggested a decoupling of leaf and air temperature in tundra environments (Lindwall et al., 2016; Potosnak et al., 2013). With a predicted increase in air temperature in the Arctic, it still remains largely unknown how leaf temperature will change and impact BVOC emissions. As suggested by Tang et al. (2016), long-term parallel observations of both leaf and air temperature are needed. The response of BVOC emissions to temperature discussed here should be interpreted with this potential caveat in mind.

**Figure 9.**
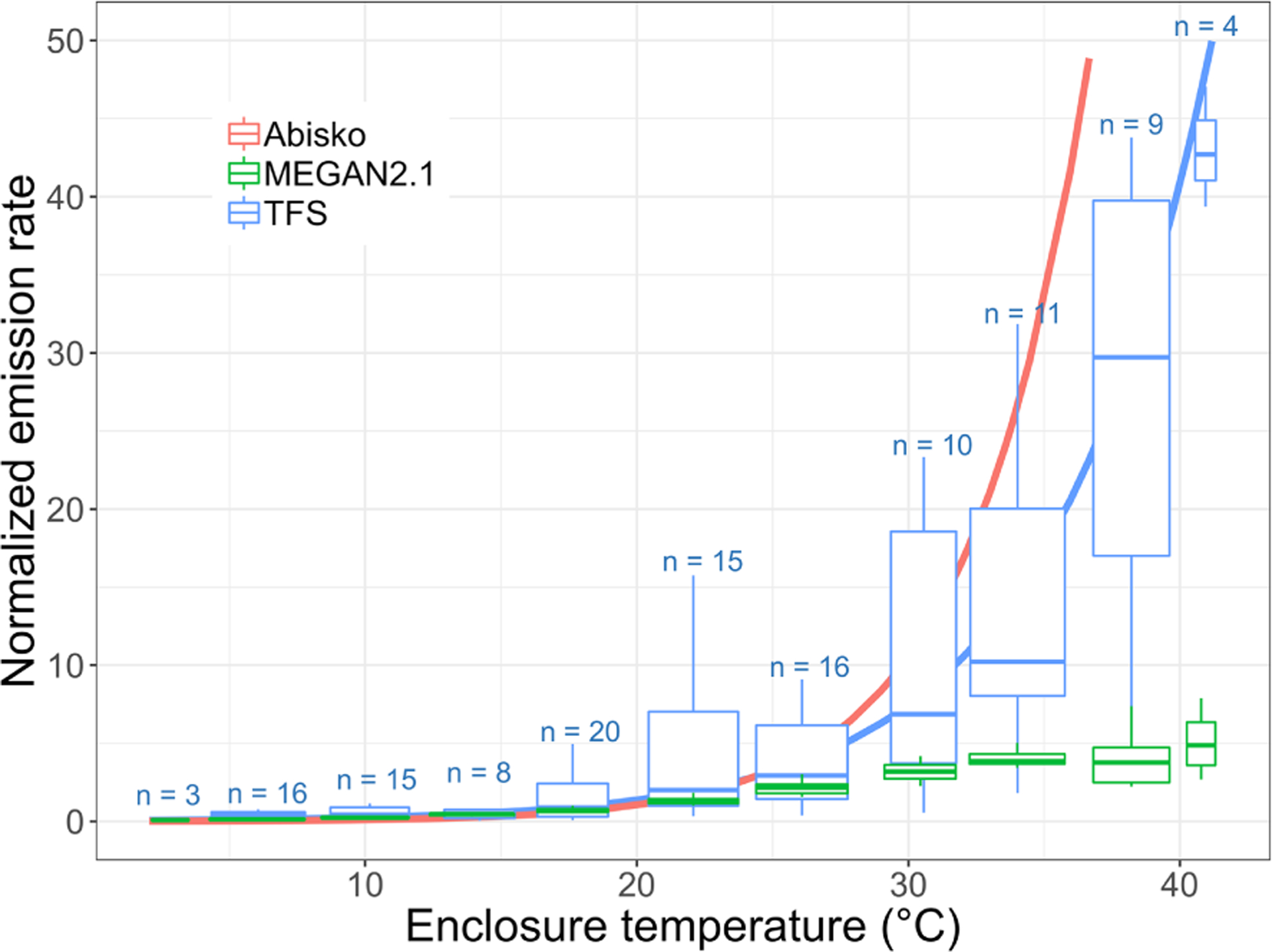
Normalized isoprene surface emission rate (emissions at 20 °C set equal to 1.0) as a function of enclosure temperature (in degrees Celsius). This figure shows the response to temperature as observed at Toolik Field Station (TFS, in blue) and Abisko, Sweden (in pink; Tang et al., 2016), and as parameterized in MEGAN2.1 (in green). The blue solid line is the exponential fit at TFS. The term *n* denotes the number of measurements in each enclosure temperature bin. It should be noted that the enclosure temperature was on average 5–6 °C warmer than ambient air due to greenhouse heating.

While MT emissions remained low and close to the detection limit, thus preventing robust quantification of any emission–temperature relationship, isoprene emissions significantly increased with temperature (Fig. 9). Figure 9 combines daytime (e.g., with relatively high PAR values) isoprene emission rates from different surface enclosures, with results normalized to account for differing total biomass and species distributions (with *Salix* spp. being the dominant emitter). Specifically, we divided all fluxes by the enclosure-specific mean emission at 20 ± 1 °C. Emission rates are often standardized to 30 °C, but we employ 20 °C here owing to the colder growth environment at TFS (Ekberg et al., 2009). The isoprene emission–temperature relationship observed at TFS (in blue) is very similar to that reported by Tang et al. (2016) at Abisko (Sweden; in pink) for tundra heath (dominated by evergreen and deciduous dwarf shrubs). Results at TFS and Abisko both point to a high isoprene temperature response for Arctic ecosystems (Tang et al., 2016). This is further supported by two warming experiments performed in mesic tundra heath (dominated by *Betula nana*, *Empetrum nigrum*, *Empetrum hermaphroditum*, and *Cassiope tetragona*) and dry dwarf shrub tundra (codominated by *Empetrum hermaphroditum* and *Salix glauca*) in western Greenland (Kramshøj et al., 2016; Lindwall et al., 2016). Kramshøj et al. (2016) observed a 240% isoprene emission increase with 3 °C warming, while Lindwall et al. (2016) reported a 280 % increase with 4 °C warming. The observationally derived emission–temperature relationship derived here for TFS reveals a 180 %–215 % emission increase with 3–4 °C warming.

The MEGAN2.1 modeling framework is commonly used to estimate BVOC fluxes between terrestrial ecosystems and the atmosphere (e.g., Millet et al., 2018). Here, we apply the TFS observations to evaluate the MEGAN2.1 emission–temperature relationship for this Arctic environment. Figure 9 shows that the model temperature algorithm provides a close fit with observations below 30 °C, with a 170 %–240 % emission increase for a 3–4 °C warming. While the model predicts a leveling-off of emissions at approximately 30–35 °C, our observations reveal no such phenomenon within the 0–40 °C enclosure temperature range (Fig. 9). However, given the limited number of enclosure measurements above 30 °C, a leveling off of emissions cannot be statistically ruled out. The key result here is that MEGAN2.1 adequately reproduces the temperature dependence response of Arctic ecosystems in the 0–30 °C temperature range, with ambient temperature > 30 °C being unlikely. The highest air temperature on record at TFS (1988–2019) is 26.5 °C, and the mean summertime (June–August) temperature over that period is 9 °C. Additionally, for each year in the 1988–2019 historical data set, there were only 1 to 23 d (0 to 4 d) per year with a maximum temperature above 20 °C (above 25 °C). If global greenhouse gas emissions continue to increase, temperatures are expected to rise 6–7 °C in northern Alaska by the end of the century (annual average; Markon et al., 2012), while the number of days with temperatures above 25 °C could triple (Lader et al., 2017). Based on current climate conditions and this rate of change, the MEGAN2.1 algorithm adequately represents the temperature dependence response of Arctic ecosystems for the near and intermediate-term future.

### 4.2 Long-term effects of warming

BVOCs produced by plants are involved in plant growth, reproduction, and defense, and plants use isoprene emissions as a thermotolerance mechanism (Peñuelas and Staudt, 2010; Sasaki et al., 2007). The exponential response of isoprene emissions to temperature observed at TFS adds to a growing body of evidence indicating a high isoprene temperature response in Arctic ecosystems. However, observations at TFS do not necessarily reflect long-term effects of warming. Schollert et al. (2015) examined how long-term warming affects the leaf anatomy of individual arctic plant shoots (*Betula nana*, *Cassiope tetragona*, *Empetrum hermaphroditum*, and *Salix arctica*). They found that long-term warming results in significantly thicker leaves, suggesting anatomical acclimation. While the authors hypothesized that this anatomical acclimation may limit the increase of BVOC emissions at plant shoot level, Kramshøj et al. (2016) later showed that BVOC emissions from Arctic tundra, exposed to 6 years of experimental warming, increase at both the plant shoot and ecosystem levels.

In addition to the direct impact of long-term warming on BVOC emissions, ecosystem level emissions are expected to increase in the Arctic due to climate-driven changes in plant biomass and vegetation composition. For instance, the widespread increase in shrub abundance in the Arctic – due to a longer growing season and enhanced nutrient availability (Berner et al., 2018; Sturm et al., 2001) – will likely significantly affect the BVOC emission potential of the Arctic tundra. Additionally, as mentioned above and as discussed extensively by Peñuelas and Staudt (2010) and Loreto and Schnitlzer (2010), emissions of BVOCs might be largely beneficial for plants, conferring them higher protection from abiotic stressors which are predicted to be more severe in the future. Long-term arctic warming may thus favor BVOC-emitting species even further.

## 5 Conclusion

While BVOC ambient concentrations and emission rates have been frequently measured in boreal ecosystems, Arctic tundra environments are under studied. We provide summertime BVOC ambient air mixing ratios and emission rates at Toolik Field Station, on the north flank of the Brooks Range in northern Alaska, here. We present the first continuous summertime record of ambient air isoprene and its first-generation oxidation products in the Arctic tundra environment. This data set provides a new set of observations to constrain isoprene chemistry in low NO_*x*_ environments. This data set also provides a baseline for investigating future changes in the BVOC emission potential of the Arctic tundra environment. While the overall mean isoprene emission rate amounted to 85 μgC m^−2^ h^−1^, elevated (> 500 μgC m^−2^ h^−1^) isoprene surface emission rates were observed for *Salix* spp., a known isoprene emitter. We also show that the response to the temperature of isoprene emissions in enclosures dominated by *Salix* spp. increased exponentially in the 0–40 °C range, likely conferring greater thermal protection for these plants. Given the widespread increase in shrub abundance in the Arctic (including *Salix* spp.), our results support earlier studies (e.g., Valolahti et al., 2015) suggesting that climate-induced changes in the Arctic vegetation composition will significantly affect the BVOC emission potential of the Arctic tundra, with implications for atmospheric oxidation processes and climate feedbacks.

## Supplementary Material

Supplementary

## Figures and Tables

**Table 1.** Year 2017 median relative percent cover of plant species in moist acidic tundra Long-Term Ecological Research (LTER) experimental control plots at Toolik Field Station. The last column indicates whether plant species were present in surface or bag enclosure experiments in this study.

Plant name	Relative land surface cover in moist acidic tundra (%) (Gough, 2019)	Present in surface or bag enclosures
*Andromeda polifolia*	0.6	Yes
*Betula nana*	14.4	Yes
*Carex bigelowii*	1.0	Yes
*Cassiope tetragona*	2.0	Yes
*Empetrum nigrum*	3.8	Yes
*Eriophorum vaginatum*	8.6	Yes
*Ledum palustre*	10.5	Yes
Mixed lichens	2.1	Yes
Mixed moss	6.0	Yes
*Pedicularis lapponica*	0.6	No
*Polygonum bistorta*	0.6	No
*Rubus chamaemorus*	20.2	No
*Salix pulchra*	4.9	Yes
*Vaccinium uliginosum*	1.9	Yes
*Vaccinium vitis-idaea*	6.6	Yes

**Table 2.** Average mixing ratios with standard deviation, along with minimum (min) and maximum (max) values and the quantification frequency (QF) of the measured monoterpenes in ambient air. LOQ stands for limit of quantification. For values lower than the LOQ, mixing ratios equal to half of the LOQ were used to calculate the mean.

	Mean ± standard deviation (pptv)	Min (pptv)	Max (pptv)	QF (%)
*α*-pinene	11.7 ± 8.1	< LOQ	61.6	88
Camphene	< LOQ	< LOQ	21.9	11
Sabinene	< LOQ	< LOQ	34.2	11
p-cymene	2.0 ± 1.9	< LOQ	12.3	32
Limonene	< LOQ	< LOQ	2.9	<1

**Table 3.** Isoprene and monoterpenes (sum of *α*-pinene, *β*-pinene, limonene, and 1,8-cineole) surface emission rates per vegetation type. Miscellaneous refers to a mix of different species, including lichens and moss tundra (see Figs. S3–S15). Daytime refers to 10:00–20:00, midday to 11:00–14:00, and nighttime to 23:00–05:00 (Alaska standard time – UTC−9). The values in parentheses represent the average enclosure temperature for each emission rate.

	Mean ± standard deviation (μgC m^−2^ h^−1^)	Daytime mean ± standard deviation (μgC m^−2^ h^−1^)	Midday mean ± standard deviation (μgC m^−2^ h^−1^)	Nighttime mean ± standard deviation (μgC m^−2^ h^−1^)
Isoprene				
*Salix* spp.	149 ± 327 (17.6 °C)	232 ± 400 (23.9 °C)	334 ± 473 (27.0 °C)	7 ± 10 (8.0 °C)
*Betula* spp.	12 ± 30 (13.7 °C)	19 ± 38 (17.4 °C)	28 ± 37 (20.1 °C)	5 ± 14 (5.8 °C)
Miscellaneous	38 ± 81 (11.8 °C)	57 ± 100 (14.8 °C)	104 ± 135 (16.2°C)	21 ± 64 (8.2 °C)
Monoterpenes				
*Salix* spp.	0.8 ± 1.3 (17.6 °C)	1.1 ± 1.5 (23.9 °C)	1.4 ± 1.7 (27.0 °C)	0.4 ± 1.0 (8.0 °C)
*Betula* spp.	0.5 ± 0.6 (13.7 °C)	0.7 ± 0.7 (17.4 °C)	1.0 ± 0.8 (20.1 °C)	0.2 ± 0.2 (5.8 °C)
Miscellaneous	1.1 ± 1.4 (11.8 °C)	1.3 ± 1.6 (14.8 °C)	1.7 ± 2.0 (16.2 °C)	1.0 ± 1.4 (8.2°C)

## Data Availability

Data are available upon request to the corresponding author.
