# Supplementary material for: Biogenic volatile organic compound ambient mixing ratios and emission rates in the Alaskan Arctic tundra
Source: Biogeosciences. Author manuscript; Available in PMC 2022 Feb 24. (PMC8872036; doi:10.5194/bg-17-6219-2020)
Supplement: Supplementary [file NIHMS1775306-supplement-Supplementary.pdf]

Supplement of Biogeosciences, 17, 6219–6236, 2020
https://doi.org/10.5194/bg-17-6219-2020-supplement
© Author(s) 2020. This work is distributed under
the Creative Commons Attribution 4.0 License.

Biogeosciences
Open Access

Supplement of

Biogenic volatile organic compound ambient mixing ratios and emission rates in the Alaskan Arctic tundra

Hélène Angot et al.

Correspondence to: Hélène Angot (helene.angot@colorado.edu)

The copyright of individual parts of the supplement might differ from the CC BY 4.0 License.

S1. Chemical analysis of enclosure and balloon samples

As described in detail elsewhere (Helmig et al. 2004; Pollmann, Ortega, and Helmig 2005), samples were thermally desorbed on an automated cartridge desorber (ATD 400, Perkin-Elmer, USA) at 300°C and injected on a GC-MS/FID (DB-1 column; 60 m × 320 µm × 5 µm). The oven temperature was set to 40°C for 2 minutes then gradually increased to 260°C at 20°C/min, and held isothermally at 260°C for 18 minutes. Note that the MS was operated in SCAN mode for the enclosure samples and in SIM mode for the balloon samples.

S1.1 Enclosure samples

The identification and quantification of the BVOC compounds were achieved by comparison with results from authentic standards from a capillary diffusion system (Helmig et al. 2003), a National Institute of Standards and Technology (NIST) certified MT standard, and using FID response factors from a well-characterized hydrocarbon standard. A set of cartridges, pre-loaded at single-digit ppb levels using the above-mentioned standards, were analyzed over a 3-month period to determine analytical uncertainties (as no certified reference material exists in equivalent matrix) and check the stability of the instrument over time. Analytical uncertainties (k = 2) were estimated to be 12.5 %, 20.0 %, 6.0 %, 12.0 %, 17.5 %, 13.0 %, 15.0 %, 10.5 %, 15.0 %, 12.5 %, 8.0 %, 17.5 %, and 11.5 % for n-pentane, isoprene, n-hexane, ethylbenzene, (m+p)-xylene, o-xylene, α-pinene, camphene, β-pinene, limonene, 1,8-cineole, isolongifolene, and α-humulene, respectively.

S1.2 Balloon samples

Isoprene (*m/z* 67 and 68) was identified and quantified using the MS in SIM mode. The response to isoprene was calibrated using a standard containing 500 ppb of methanol, acetone, and isoprene (Apel-Riemer Environmental Inc., Miami, FL, USA). The peak area of CFC-113 (*m/z* 85, 101, 103, and 151) was used to correct for sample volume variations in the cartridges.

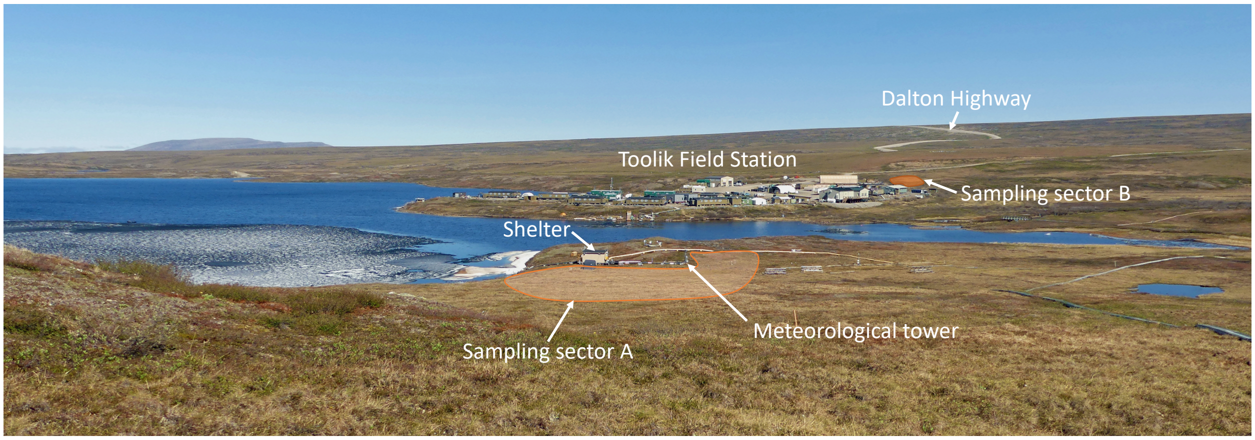

Figure S1: Photograph of the area around Toolik Field Station. Ambient air measurements where performed in the instrument shelter located across the lake. Emission rates where measured in sampling sectors A and B.

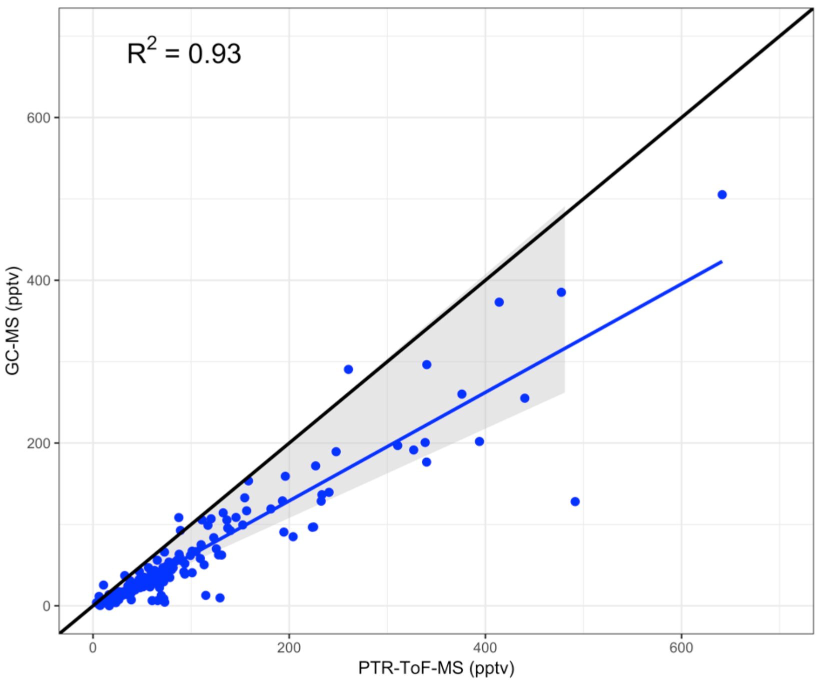

Figure S2: Comparison of isoprene mixing ratios in ambient air by gas chromatography and mass spectrometry (GC-MS; y-axis) and by proton-transfer reaction time-of-flight mass spectrometry (PTR-ToF-MS; x-axis) during the summer 2019 campaign. The grey shaded region gives the uncertainty on the regression line (in blue) based on the analytical uncertainty for isoprene of the two instruments. The black line is the bisector.

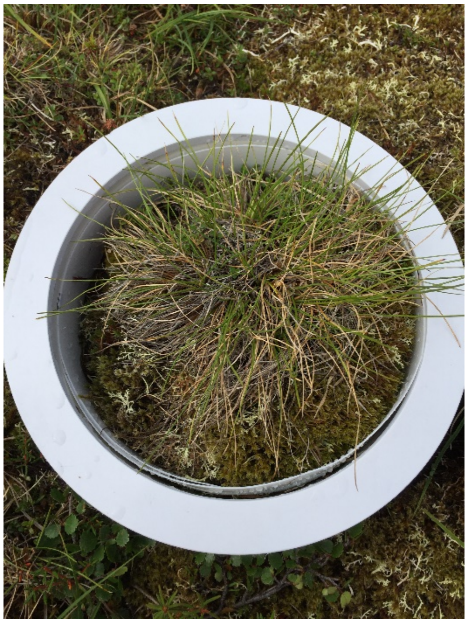

Figure S3: Enclosure set up on July 20, 2018 and sampled from July 21 to 23, 2018; *Eriophorum vaginatum*, lichens, mosses. Vegetation type: **Miscellaneous**

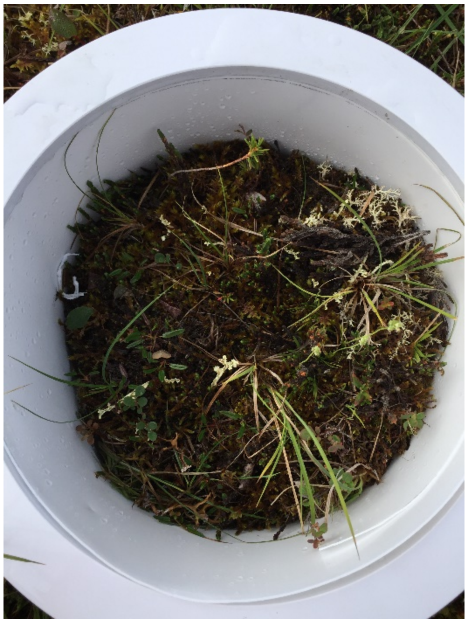

Figure S4: Enclosure set up on July 23, 2018 and sampled from July 24 to 26, 2018; *Dryas integrifolia*, *Liverwort*, *Rhododendron tomentosum* (formerly known as *Ledum palustre*), *Tofieldia*, *Tomentypnum nitens* (moss), *Carex bigelowii*, *Cassiope tetragona*, *Vaccinium uliginosum*. Vegetation type: **Miscellaneous**

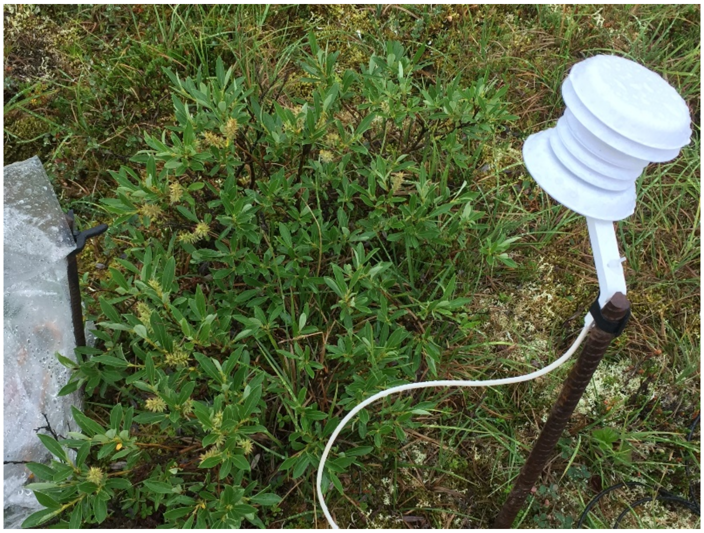

Figure S5: Branch enclosure set up on July 26, 2018 and sampled from July 27 to August 2, 2018; *Salix glauca* (willow). Vegetation type: **Salix spp.**

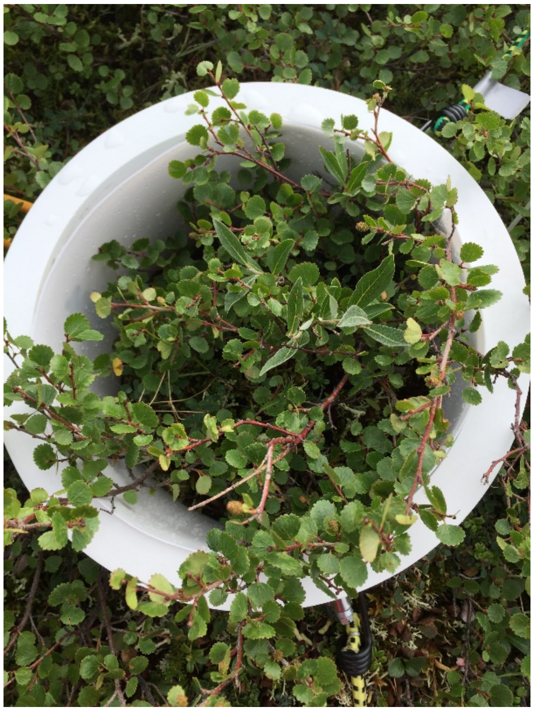

Figure S6: Enclosure set up on July 21, 2018 and sampled from July 22 to 25, 2018; *Betula nana, Empetrum nigrum* (ericaceous shrub), *Vaccinium vitis-idaea* (short evergreens shrub), *Rhytidium Rugosum* (bryophyte), a branch of *Salix glauca*. Vegetation type: **Betula spp.**

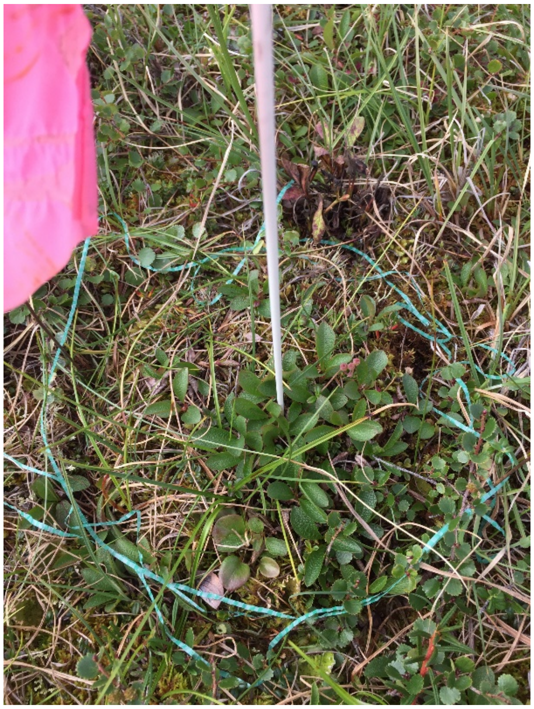

Figure S7: Enclosure set up on July 25, 2018 and sampled from July 26-28, 2018; *Arctostaphylos alpina, Andromeda polifolia, Betula nana, Carex bigelowii, Vaccinium vitis-idaea, Dicranum* (moss). Vegetation type: **Betula spp.**

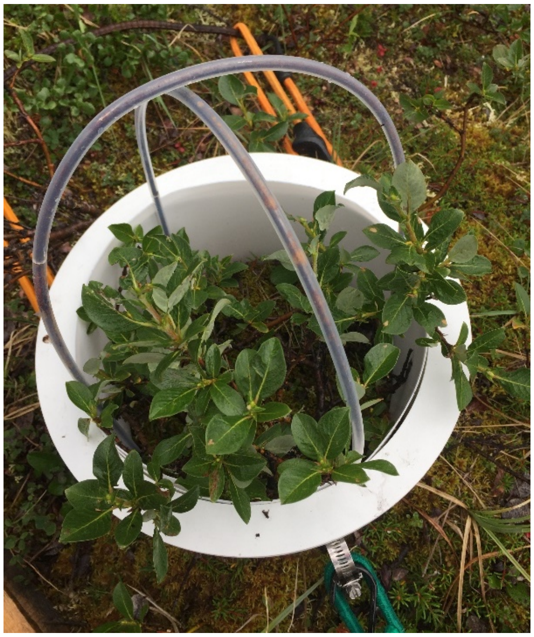

Figure S8: Enclosure set up on July 28, 2018 and sampled from July 29 to August 1, 2018; *Salix pulchra, Hylocomium splendens, Rhododendron tomentosum* (formerly known as *Ledum palustre*), *Vaccinium vitis-idaea.* Vegetation type: **Salix spp.**

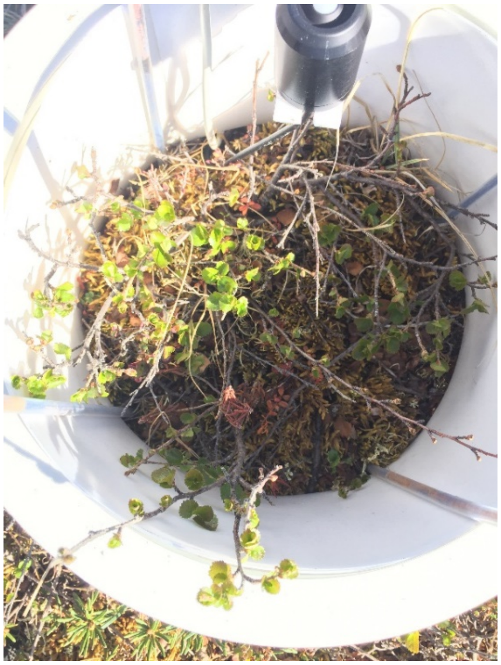

Figure S9: Enclosure set up on June 4, 2019 and sampled from June 5-12, 2019; *Betula nana*, moss. Vegetation type: **Betula spp.**

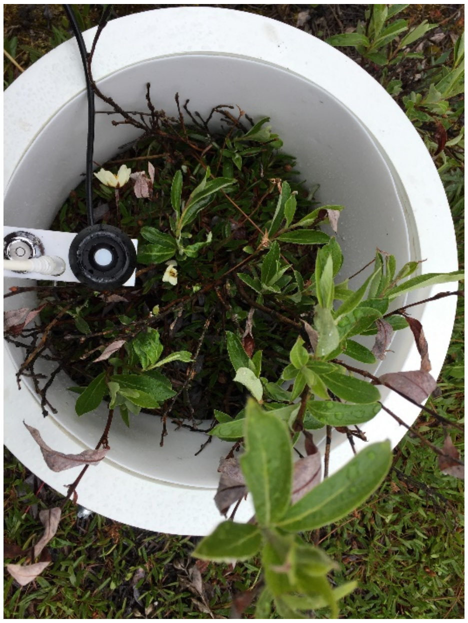

Figure S10: Enclosure set up on June 14, 2019 and sampled from June 15-18, 2019; *Dryas integrifolia*, *Salix pulchra*. Vegetation type: **Salix spp.**

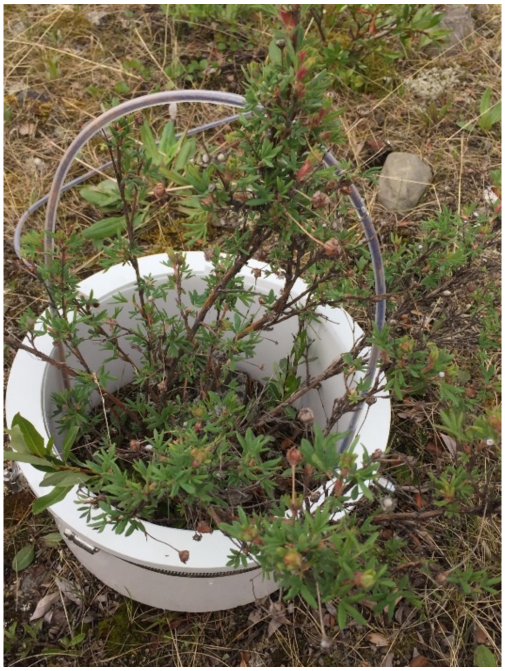

Figure S11: Enclosure set up on June 18, 2019 and sampled from June 19-27, 2019; *Rhododendron tomentosum* (formerly known as *Ledum palustre*), *Salix pulchra*, *Potentilla fruticosa*. Vegetation type: **Salix spp.**

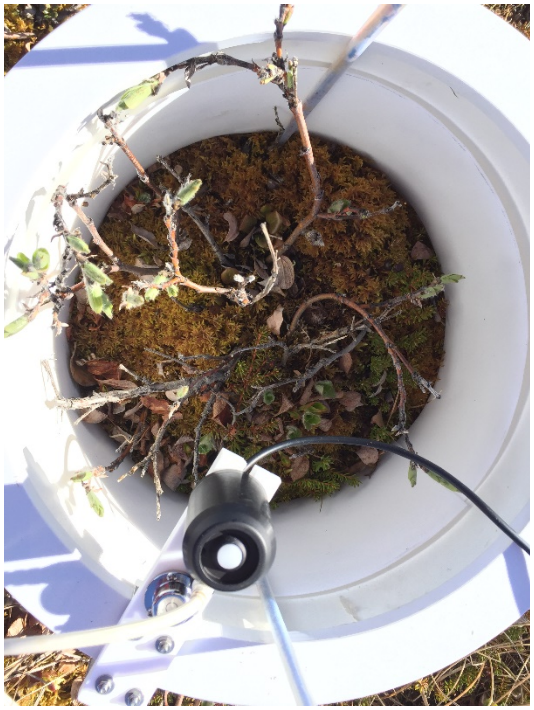

Figure S12: Enclosure set up on May 30, 2019 and sampled from June 3-4, 2019; *Empetrum nigrum*, *Arctostaphylos alpina, Salix glauca*, *Hylocomium splendens*. Vegetation type: **Salix spp.**

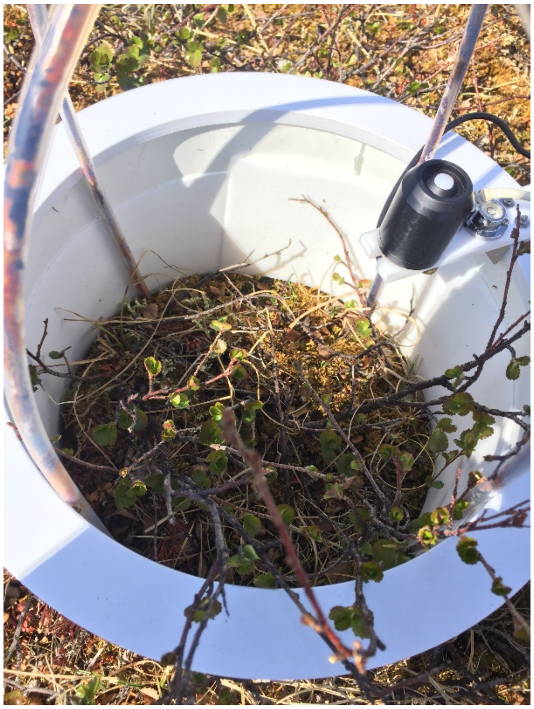

Figure S13: Enclosure set up on June 4, 2019 and sampled from June 5-9, 2019; *Betula nana*, *Carex bigelowii, Hylocomium splendens*. Vegetation type: **Betula spp.**

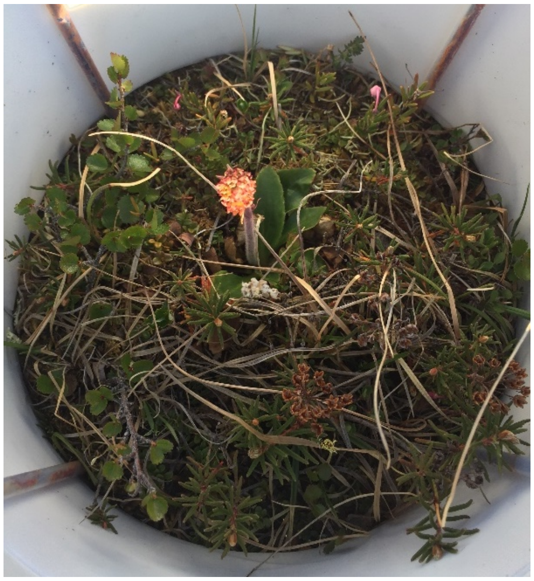

Figure S14: Enclosure set up on June 9, 2019 and sampled from June 10-12, 2019; *Betula nana*, *Carex bigelowii*, *Vaccinium vitis-idaea*, *Andromeda polifolia, Saxifraga heiracifolia*. Vegetation type: **Miscellaneous**

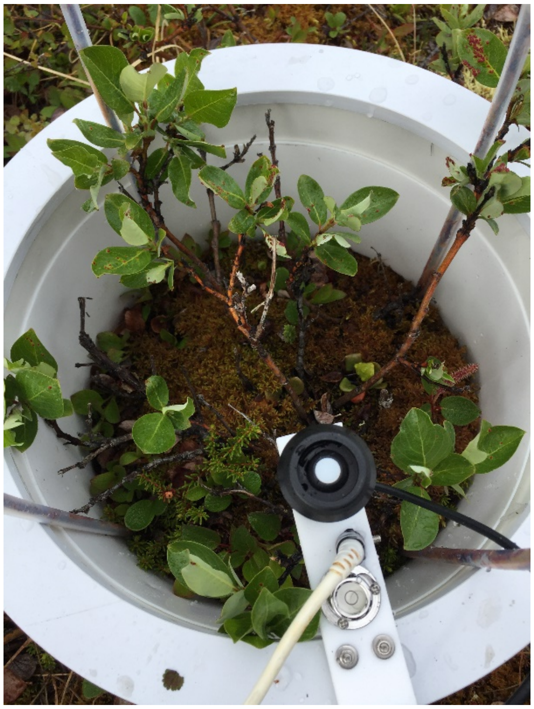

Figure S15: Enclosure set up on June 14, 2019 and sampled from June 15-25, 2019; *Salix chamissonis*, *Salix reticulata, Empetrum nigrum*. Vegetation type: **Salix spp.**

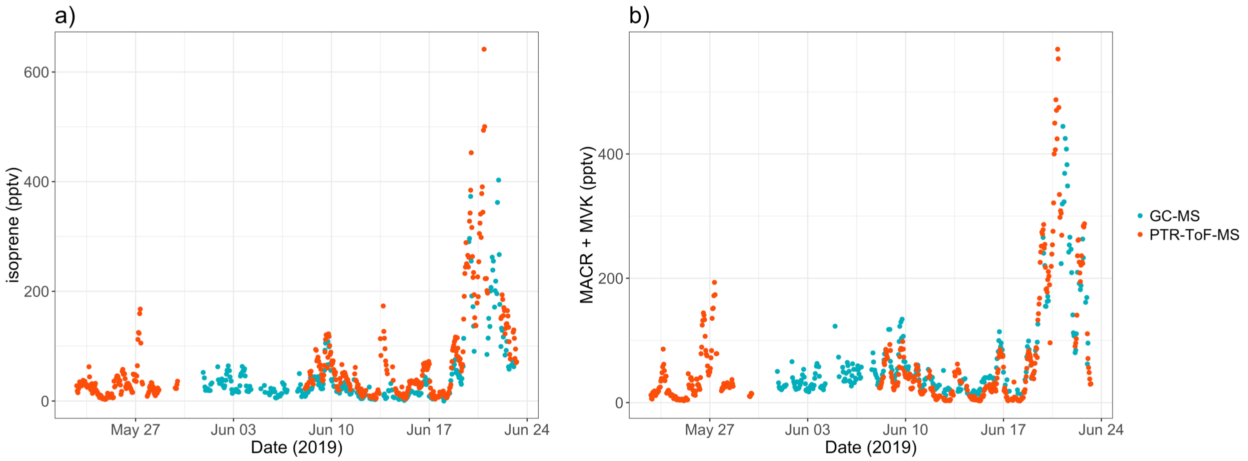

Figure S16: Time-series of a) isoprene and b) methylvinylketone (MVK) plus methacrolein (MACR) mixing ratios in ambient air at Toolik Field station by gas chromatography and mass spectrometry (GC-MS, in blue) and proton-transfer reaction time-of-flight mass spectrometry (PTR-ToF-MS, in orange) during the 2019 field campaign.

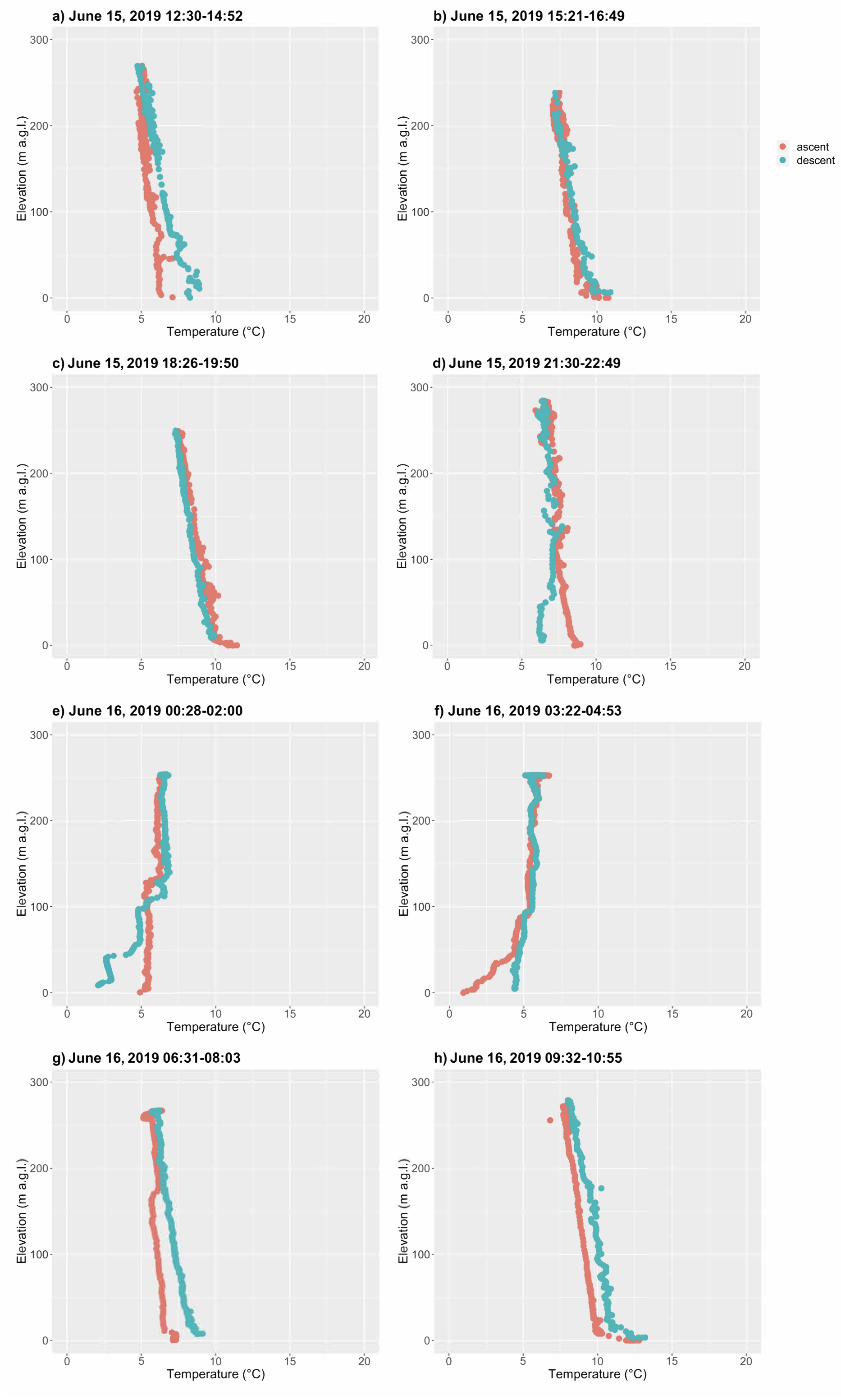

Figure S17: Temperature vertical profiles during balloon flights on June 15 and 16, 2019. Hours are in Alaska Standard Time (UTC-9). The pink and blue dots correspond to the ascent and descent of the tethered balloon, respectively.

References

Helmig, Detlev, Florence Bocquet, Jan Pollmann, and Tobias Revermann. 2004. "Analytical Techniques for Sesquiterpene Emission Rate Studies in Vegetation Enclosure Experiments." *Atmospheric Environment* 38 (4): 557–72. https://doi.org/10.1016/j.atmosenv.2003.10.012.

Helmig, Detlev, Tobias Revermann, Jan Pollmann, Oliver Kaltschmidt, Aidaris Jiménez Hernández, Florence Bocquet, and Donald David. 2003. "Calibration System and Analytical Considerations for Quantitative Sesquiterpene Measurements in Air." *Journal of Chromatography A* 1002 (1): 193–211. https://doi.org/10.1016/S0021-9673(03)00619-8.

Pollmann, Jan, John Ortega, and Detlev Helmig. 2005. "Analysis of Atmospheric Sesquiterpenes: Sampling Losses and Mitigation of Ozone Interferences." *Environmental Science & Technology* 39 (24): 9620–29. https://doi.org/10.1021/es050440w.
